# An interaction between OTULIN and SCRIB uncovers roles for linear ubiquitination in planar cell polarity

**DOI:** 10.1242/dmm.049762

**Published:** 2023-08-17

**Authors:** Stephanie M. Almeida, Sofiia Ivantsiv, Rieko Niibori, Wade H. Dunham, Brooke A. Green, Liang Zhao, Anne-Claude Gingras, Sabine P. Cordes

**Affiliations:** ^1^ Lunenfeld-Tanenbaum Research Institute, Mount Sinai Hospital, 600 University Ave, Toronto, ON M5G 1X5, Canada; ^2^ Department of Molecular Genetics, University of Toronto, Toronto, ON M5S 1A8, Canada

**Keywords:** Linear ubiquitin assembly complex, OTULIN, Scribble, Linear ubiquitin, Mouse genetics, Planar cell polarity

## Abstract

Planar cell polarity (PCP) plays critical roles in developmental and homeostatic processes. Membrane presentation of PCP complexes containing Van Gogh-like (VANGL) transmembrane proteins is central to PCP and can be directed by the scaffold protein scribble (SCRIB). The role atypical linear ubiquitin (Met1-Ub) chains might play in PCP is unknown. Here, HEK293 cell-based interactomic analyses of the Met1-Ub deubiquitinase OTULIN revealed that OTULIN can interact with SCRIB. Moreover, Met1-Ub chains associated with VANGL2 and PRICKLE1, but not SCRIB, can direct VANGL2 surface presentation. Mouse embryos lacking *Otulin* showed variable neural tube malformations, including rare open neural tubes, a deficit associated with PCP disruption in mice. In Madin–Darby canine kidney cells, in which the enrichment of VANGL2-GFP proteins at cell-cell contacts represents activated PCP complexes, endogenous OTULIN was recruited to these sites. In the human MDA-MB-231 breast cancer cell model, OTULIN loss caused deficits in Wnt5a-induced filopodia extension and trafficking of transfected HA-VANGL2. Taken together, these findings support a role for linear (de)ubiquitination in PCP signaling. The association of Met1-Ub chains with PCP complex components offers new opportunities for integrating PCP signaling with OTULIN-dependent immune and inflammatory pathways.

## Teaser

**Summary:** OTULIN interacts with SCRIB, can regulate the association of linear ubiquitin chains with planar cell polarity (PCP) components and, in MDA-MB-231 cells, affects hallmark PCP features, including Wnt5a-induced filopodia extension and VANGL2 trafficking.

## INTRODUCTION

The regulated conjugation and removal of ubiquitin chains can control protein activities, localization and degradation, and thereby play key roles in development, cellular homeostasis and disease. Although, typically, ubiquitin chains are built by attaching a ubiquitin moiety to one of seven lysines present within a recipient ubiquitin and result in K6, K11, K21, K27, K36, K48 or K63 chains (Kirisako et al., 2006; reviewed in Spit et al., 2019; Weinelt and van Wijk, 2021); less commonly, ubiquitin molecules can be linked head-to-tail to form linear ubiquitin chains (N-terminal methionine-linked ubiquitin or Met1-Ub). In vertebrates, only the linear ubiquitin assembly complex (LUBAC) is known to construct Met1-Ub chains (Behrends and Harper, 2011; Tokunaga et al., 2011; Walczak et al., 2012). LUBAC consists of three proteins: heme-oxidized IRP2 ubiquitin ligase 1 (HOIL-1, gene name *RBCK1*), the catalytic subunit HOIL-1-interacting protein (HOIP, gene name *RNF31*) and SHANK-associated RH-domain-interacting protein (SHARPIN) (Kirisako et al., 2006; Tokunaga et al., 2009, 2011; Gerlach et al., 2011; Ikeda et al., 2011). Deubiquitination, the removal of ubiquitin, is equally important (Nijman et al., 2005). Ovarian tumor deubiquitinase with linear linkage specificity (OTULIN), also called GUMBY or FAM105B, emerged in (uro)chordates to selectively disassemble Met1-Ub chains (Keusekotten et al., 2013; Rivkin et al., 2013). LUBAC and OTULIN are physically and functionally intertwined. A PNGase/UBA or UBX (PUB) domain present in HOIP directly binds a five-amino-acid PUB domain-interacting motif (PIM) located N-terminal to the Met1-Ub chain-cleaving ovarian tumor (OTU) domain of OTULIN (Elliott et al., 2014; Schaeffer et al., 2014; Takiuchi et al., 2014). OTULIN activity reverses LUBAC-dependent effects by removing Met1-Ub chains from client proteins but can also activate LUBAC by removing auto-inhibitory Met1-Ub chains from LUBAC components (Heger et al., 2018). Thus, OTULIN helps to exquisitely balance Met1-Ub homeostasis.

The molecular programs that are selected for and regulated by linear (de)ubiquitination are still emerging. However, in examples examined to date, target pathway selection has been determined by LUBAC components. So, for instance, the binding of HOIP to a polyubiquitin platform containing K63 chains attached to the tumor necrosis factor (TNF) receptor signaling complex (TNF-RSC) recruits LUBAC (Haas et al., 2009). Then HOIP, via its PUB domain, draws OTULIN to TNF-RSC. In this and other cases, HOIP drives the choice of the signaling complex and its associated components for Met1-Ub conjugation. A case wherein OTULIN might be the leading actor in target pathway choice has not been identified.

Wnt signaling regulates an array of developmental and adult homeostatic processes, as illustrated by the embryonic patterning defects and increased tissue-specific inflammation and cancer incidence that arise in mice and humans upon its disruption (Loh et al., 2016). Wnts can activate numerous intracellular signaling cascades, broadly divided into the canonical β-catenin-dependent pathway and the non-canonical β-catenin-independent pathways, which include the planar cell polarity (PCP) pathway. OTULIN can interact with Dishevelled 2 (Dvl2) (Rual et al., 2005; Rivkin et al., 2013), an integrator of Wnt signaling pathways. In cell culture, LUBAC inhibits the expression of a β-catenin-dependent reporter and OTULIN can reverse this inhibition (Rivkin et al., 2013; Takiuchi et al., 2014). In MDA-MB-231 breast cancer cells, these effects rely on degradation of Met1-Ub-modified β-catenin (Wang et al., 2020). Whether LUBAC and OTULIN modify components dedicated to other Wnt signaling pathways, such as the PCP pathway, and regulate these pathways has not been explored.

During PCP signaling, core PCP proteins, which include Van Gogh-like proteins 1 and 2 (VANGL1 and VANGL2), Dishevelled 1-3 (Dvl1-3), Prickle (Pk in *Drosophila*, PRICKLE1 in vertebrates) and the Frizzled receptors (Fzd) (Seifert and Mlodzik, 2007; Wang and Nathans, 2007), are distributed in an asymmetrical manner at the cell surface to direct cellular orientation (reviewed in Yang and Mlodzik, 2015; Lavalou and Lecuit, 2022). A critical event in PCP signaling is the surface delivery of the four-transmembrane-domain-containing VANGL1 and VANGL2 proteins (reviewed in Bailly et al., 2018; Dreyer et al., 2022), which relies in part on interactions mediated by their PDZ-binding motifs (PBMs) (Kim et al., 1995). PBMs are short, canonical, usually C-terminal sequences, which bind to specific PDZ domains (Songyang et al., 1997; Giallourakis et al., 2006; Stiffler et al., 2007; te Velthuis et al., 2011). VANGL2 contains two PBMs: a canonical C-terminal type I PBM, which interacts with the peripheral membrane and scaffolding protein scribble (SCRIB), and an unconventional internal PBM, which binds Dvl2 and is mutated in the *Vangl2^S464N^* [also called *Looptail* (*Lp*)] mouse mutant (Torban et al., 2004b). Loss of the Dvl2-VANGL2 interaction in *Vangl2^S464N^* mice or of the VANGL PBM-interacting PDZ domains of SCRIB in *Scrib1^Crc/Crc^* [also called *Circletail* (*Crc*)] mice leads to PCP defects, including failure of neural tube closure (Montcouquiol et al., 2003; Torban et al., 2004a). Likewise, mutations in *VANGL1* (Kibar et al., 2009), *VANGL2* (Kibar et al., 2009, 2011; Gravel et al., 2010) and *SCRIB* (Lei et al., 2013) have been identified in familial and sporadic cases of spina bifida in humans, and *PRICKLE1* mutations have been associated with epilepsy and neural tube defects (Bassuk et al., 2008; Bosoi et al., 2011; Hata et al., 2019).

Here, our analyses of the OTULIN interactome revealed that OTULIN, via its PBM, interacts with SCRIB. SCRIB directs apical-basal polarity and PCP by organizing the distribution of distinct, mutually antagonistic protein complexes. Here, we focused on the possible impact of OTULIN on SCRIB and the core PCP component VANGL2. In HEK293 cells, we found that VANGL2 and PRICKLE1, but not SCRIB, might be conjugated to Met1-Ub chains. Met1-Ub modification of VANGL2 correlated with its surface presentation, whereas catalytically active OTULIN promoted VANGL2 internalization. Subsequently, we examined OTULIN and its effect on PCP hallmarks in several established PCP cell culture model systems. In Madin–Darby canine kidney (MDCK) cells, in which expression of GFP-VANGL1 or GFP-VANGL2 proteins is thought to drive the formation of activated PCP complexes at cell-cell contacts, endogenous OTULIN was recruited to these sites. Mouse embryos lacking *Otulin* showed neural tube malformations, ranging from collapsed roof plates to, more rarely, open neural tubes. Finally, we found that in the MDA-MB-231 breast cancer cell model for Wnt5a signaling, OTULIN loss led to deficits in Wnt5a-induced filopodia extension and redistribution of transfected HA-VANGL2. Taken together, the disruption of hallmark features in multiple PCP model systems supports an important role for linear ubiquitin homeostasis in optimal PCP signaling.

## RESULTS

### Identification of an OTULIN-SCRIB interaction with a possible role in PCP signaling

**Fig. 1.**
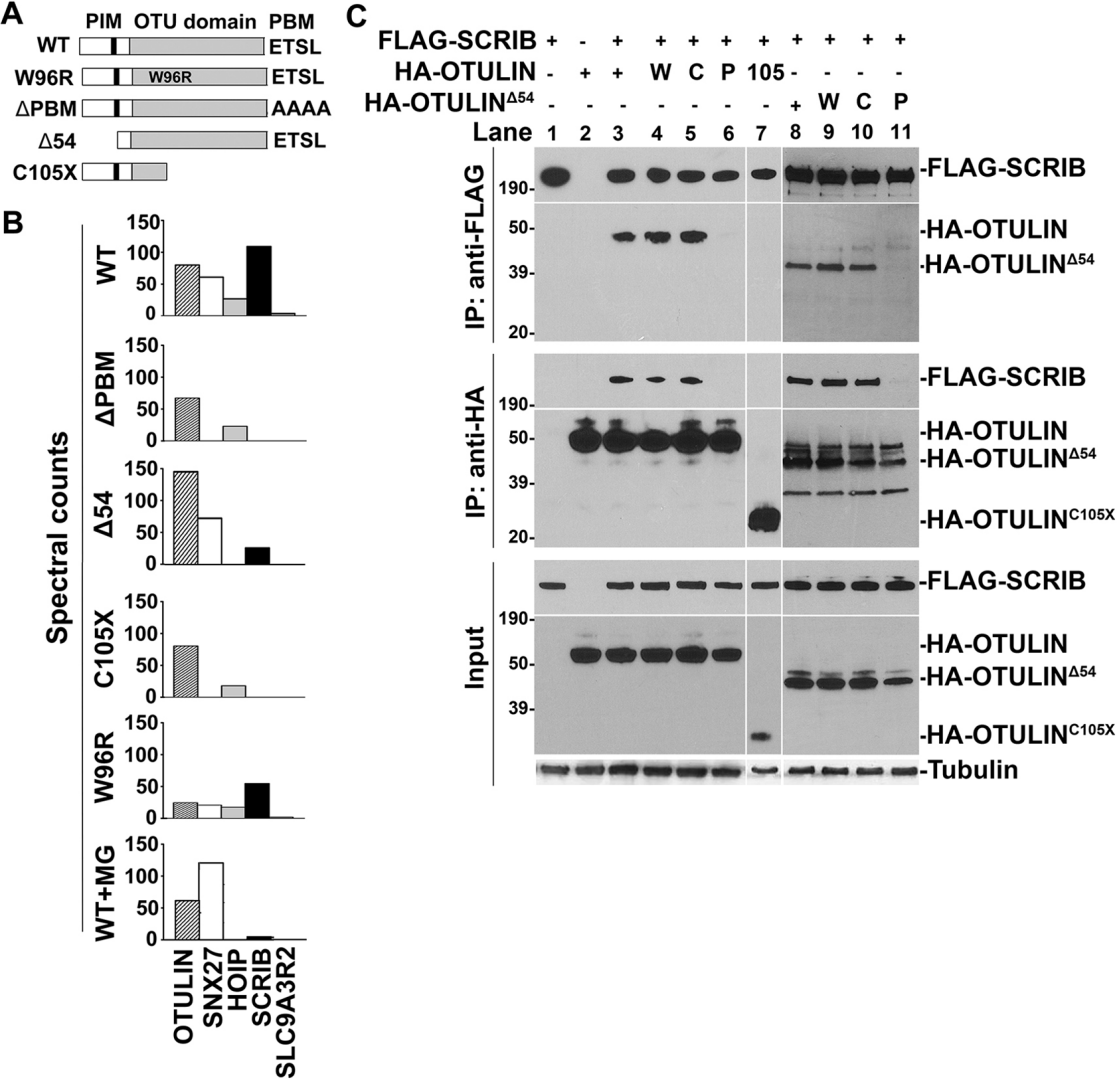
**OTULIN uses its PBM to interact with SCRIB.** (A) Schematic diagram showing FLAG-tagged OTULIN constructs used in AP-MS experiments: full-length OTULIN (WT), catalytically inactive OTULIN (W96R), OTULIN lacking its PBM (ΔPBM) or N-terminal 54 amino acids (Δ54), and OTULIN truncated after amino acid 105 (C105X). PIM, PUB domain-interacting motif; PBM, PDZ domain-binding motif. (B) Spectral counts recovered in AP-MS experiments. OTULIN^ΔPBM^ did not recover SNX27, SCRIB or SLC9A3R2. The N-terminal region of OTULIN is required for its interactions with HOIP (Rivkin et al., 2013). Notably, reduced SCRIB and no HOIP peptides were recovered by full-length OTULIN treated with the proteasomal inhibitor MG132 (WT+MG) or by OTULIN^Δ54^. (C) Immunoprecipitation (IP) of FLAG-SCRIB with an anti-FLAG antibody recovered equivalent levels of WT HA-OTULIN, HA-OTULIN^W96R^ (‘W’) and HA-OTULIN^C129S^ (‘C’), as detected by immunoblotting with an anti-HA antibody. Conversely, immunoprecipitation of HA-OTULIN using an anti-HA antibody recovered FLAG-SCRIB when the OTULIN PBM was present, but this was not seen for HA-OTULIN^ΔPBM^ constructs (‘P’). Deletion of the N-terminal HOIP-interacting region (HA-OTULIN^Δ54^ constructs) did not affect interactions with SCRIB. HA-OTULIN^C105X^ (‘105’) did not interact with FLAG-SCRIB. Input amounts for each construct and tubulin levels are shown. Immunoblots are representative of three independent experiments.

To gain insights into the biological roles of OTULIN, we performed affinity purification (AP) of FLAG-tagged OTULIN and its associated proteins, followed by mass spectrometry (MS). Full-length OTULIN possesses a PBM and, when expressed in HEK293 cells, interacts with three PDZ-domain-containing proteins: the PCP protein SCRIB, the endosomal sorting protein SNX27 and, to a lesser degree, SLC9A3R2, also called NHERF2 (Fig. 1A,B;  Table S1). OTULIN constructs containing a mutated PBM consisting of four sequential alanine residues (OTULIN^ΔPBM^) or only the first 105 N-terminal amino acids of OTULIN (OTULIN^C105X^) did not retrieve SCRIB, SNX27 or SLC9A3R2. SNX27 and SLC9A3R2 have been reported in other OTULIN interactomic analyses (Stangl et al., 2019; Huttlin et al., 2021; Shi et al., 2021). Confirming the reliance of the OTULIN-SCRIB interaction on the OTULIN PBM motif: HA-OTULIN and FLAG-SCRIB co-immunoprecipitated only when HA-OTULIN constructs harbored an intact PBM (Fig. 1C). When deubiquitinase activity was compromised by using FLAG-OTULIN^W96R^ in AP-MS analyses (Fig. 1B; Table S2) and HA-OTULIN^W96R^ or HA-OTULIN^C129S^ in co-immunoprecipitation experiments (Fig. 1C), OTULIN-SCRIB interactions were not disrupted.

These interactomics analyses also suggest that other unknown mechanisms may regulate the stability of the OTULIN-SCRIB interaction. The presence of an intact PIM motif in the N-terminus of OTULIN increases the recovery of endogenous SCRIB, as AP-MS experiments with the full-length wild-type (WT) OTULIN consistently recovered more SCRIB peptides than a bait lacking this sequence (OTULIN^Δ54^, which lacks the first 54 N-terminal amino acids of OTULIN) (Fig. 1B; Table S1). Overexpression of FLAG-SCRIB overcame this effect as HA-OTULIN and HA-OTULIN^Δ54^ recovered FLAG-SCRIB equivalently in co-immunoprecipitation experiments when both the bait and prey were transiently expressed. Interestingly, in AP-MS experiments, proteasomal inhibition with MG132 abrogated recovery of the OTULIN-HOIP interaction and also reduced recovery of the OTULIN-SCRIB interaction (Fig. 1B; Table S2), suggesting that other, less stable proteins can interfere with both HOIP-OTULIN and OTULIN-SCRIB complexes. Thus, fundamentally, the OTULIN-SCRIB interaction requires the PBM of OTULIN, but its stability may be increased by its PIM-containing region and other proteins.

**Fig. 2.**
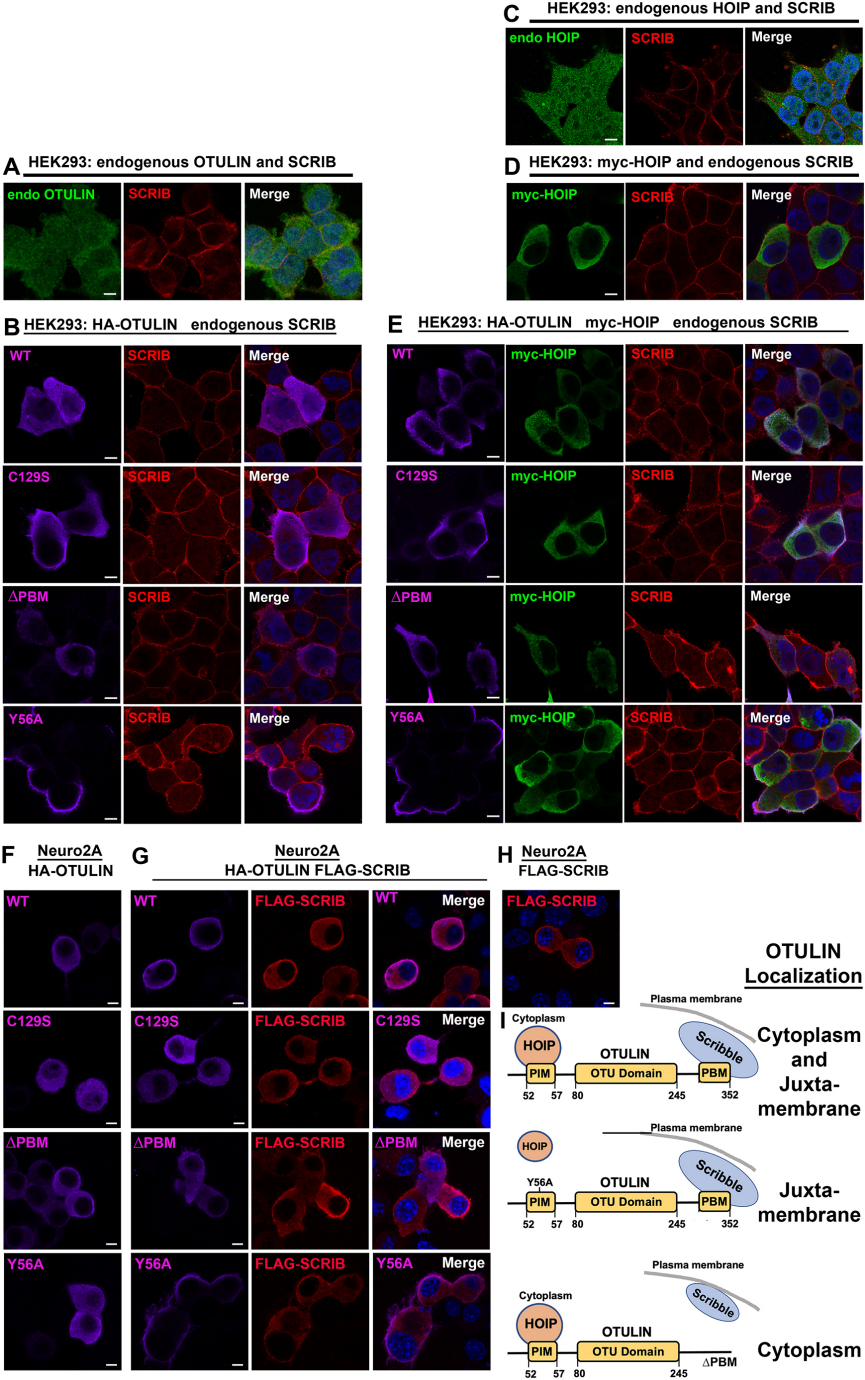
**Localization of OTULIN to juxtamembrane positions depends on its PBM and SCRIB.** (A-E) Representative images show immunofluorescence staining of endogenous SCRIB (red) in HEK293 cells co-stained for (A) endogenous (‘endo’) OTULIN (green); (B) transfected HA-OTULIN constructs (magenta) – HA-OTULIN (WT), HA-OTULIN^C129S^ (C129S), HA-OTULIN^ΔPBM^ (no PBM) and HA-OTULIN^Y56A^ (Y56A); (C) endogenous HOIP (green); (D) transfected myc-HOIP (green); and (E) co-transfected myc-HOIP (green) and HA-OTULIN constructs (WT, C129S, ΔPBM and Y56A) (magenta). (F-H) Representative images show immunofluorescence staining of Neuro2A cells (F) transfected with HA-OTULIN constructs (WT, C129S, ΔPBM and Y56A) (magenta), (G) co-transfected with FLAG-SCRIB (red) and HA-OTULIN constructs (magenta), and (H) transfected with FLAG-SCRIB (red). (I) A schematic diagram summarizes subcellular localization of HA-OTULIN constructs. Images are representative of three independent experiments. Scale bars: 10 µm.

Next, we explored the impact of SCRIB and OTULIN on each other's subcellular localization. Endogenous SCRIB was enriched near the plasma membrane of HEK293 cells (Fig. 2A). Endogenous OTULIN, transfected HA-OTULIN and catalytically compromised HA-OTULIN^C129S^ were all found predominantly in the cytoplasm, with enrichment at the plasma membrane periphery (Fig. 2B). Removing the OTULIN PBM (HA-OTULIN^ΔPBM^) resulted in cytoplasmic distribution without apparent juxtamembrane enrichment. Strikingly, HA-OTULIN^Y56A^, which specifically cannot bind HOIP (Schaeffer et al., 2014), localized to the plasma membrane periphery but was excluded from the central cytoplasmic region of all transfected cells (Fig. 2B). Co-transfection of myc-HOIP, which is expressed throughout the cytoplasm (Fig. 2C,D), did not alter the localization of any HA-OTULIN constructs (Fig. 2E). Neither HA-OTULIN constructs nor myc-HOIP affected SCRIB localization.

To further test whether SCRIB might recruit OTULIN to the plasma membrane periphery, we examined the expression of HA-OTULIN proteins in mouse Neuro2A (N2A) cells, in which appreciable levels of endogenous SCRIB were not detected (Fig. 2F-H). In contrast to their expression in HEK293 cells, all HA-OTULIN proteins, when transfected alone, showed equivalent broad cytoplasmic distribution (Fig. 2F). However, upon co-transfection with FLAG-SCRIB, which is enriched at the plasma membrane periphery (Fig. 2G,H), the subcellular localization of HA-OTULIN constructs recapitulated that seen in HEK293 cells (Fig. 2G): HA-OTULIN and HA-OTULIN^C129S^ showed cytoplasmic expression with enrichment near the membrane, HA-OTULIN^ΔPBM^ showed cytoplasmic expression without juxtamembrane enrichment and HA-OTULIN^Y56A^ showed juxtamembrane localization exclusively. These observations were consistent with SCRIB recruiting OTULIN to juxtamembrane locations, whereas PIM-dependent interactions of OTULIN – most likely with HOIP – were responsible for OTULIN localization to other cytoplasmic regions and/or organelles (Fig. 2I).

### Optimal OTULIN-Dvl2 interaction requires the PCP signaling-associated DEP domain of Dvl2

Orthogonal support for the possible involvement of OTULIN in PCP signaling arose from our probing of the established OTULIN-Dvl2 interaction, which does not rely on the OTULIN PBM (Rivkin et al., 2013; Takiuchi et al., 2014) (Fig. S1). Dvl proteins harbor three conserved domains: the self-oligomerizing amino-terminal DIX domain, a central PDZ domain, and a C-terminal DEP domain. Each of these domains functions uniquely in Wnt pathways. Dvl2 transduces canonical Wnt signaling via the DIX domain. The PCP pathway utilizes the Dvl2 PDZ domain to interact with VANGL1 and VANGL2. The DEP domain is used for membrane anchoring required for PCP signaling. Reciprocal co-immunoprecipitation assays in HEK293T cells revealed that removing DIX or PDZ domains did not reduce the recovery of FLAG-OTULIN/Dvl2 complexes (Fig. S1B). However, loss of the DEP domain diminished the Dvl2-OTULIN interaction, suggesting that OTULIN associates directly or indirectly with a fraction of membrane-tethered Dvl2 and that OTULIN might play a role in PCP signaling.

### Endogenous OTULIN co-localizes with PCP complexes in the MDCK model

**Fig. 3.**
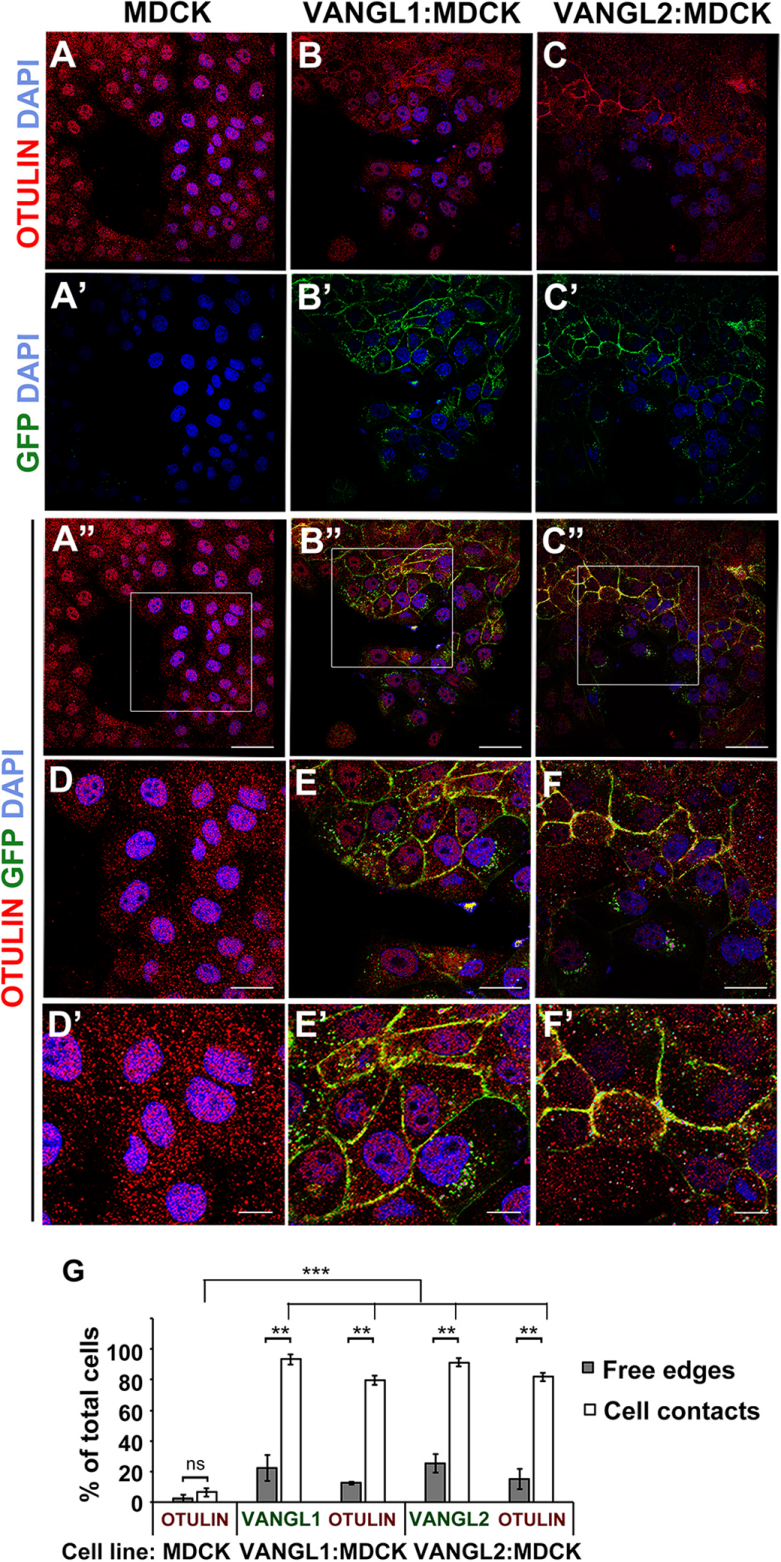
**Endogenous OTULIN is recruited to GFP-VANGL1- and GFP-VANGL2-containing PCP complexes in MDCK cells.** (A-A″,D,D′) In untransduced MDCK cells, endogenous OTULIN expression is punctate and distributed uniformly throughout the cell. (B-C″,E-F′) MDCK cells transduced with (B-B″,E,E′) GFP-VANGL1 or (C-C″,F,F′) GFP-VANGL2 display characteristic VANGL1/2 localization to the cell membrane at sites of cell-cell contact when cells are grown to 80% confluency. Endogenous OTULIN is enriched at these sites where GFP-VANGL1 or GFP-VANGL2 is co-localized but not at cell-cell contacts of untransduced cells. D-F show higher magnification views of outlined regions shown in A″-C″, respectively. Images are representative of three independent experiments. Scale bars: 50 µm (A-C″); 25 µm (D-F); 10 µm (D′-F′). (G) Quantification of the percentage of total cells with endogenous OTULIN at free edges or localized to edges in contact with neighboring cells is shown for untransduced MDCK cells (MDCK), and GFP-VANGL1 (VANGL1:MDCK) and GFP-VANGL2 (VANGL2:MDCK) transduced MDCK cells. Error bars show the s.e.m. Statistical significance values between the localization of OTULIN in untransduced cells compared to GFP-VANGL1- or GFP-VANGL2-expressing cells were determined using two-way ANOVA and the Bonferroni post test. ns, not significant; **P<0.001; ***P<0.0001.

SCRIB regulates apical-basal polarity and PCP by associating with distinct partner proteins (reviewed in Bonello and Peifer, 2019). Given our interest in PCP signaling, we next asked whether endogenous OTULIN might be recruited from the cytoplasm to known SCRIB-containing PCP signaling complexes in MDCK cells, in which the stably expressed core PCP proteins VANGL1 and VANGL2 (tagged with GFP) co-localize with endogenous SCRIB to sites of cell-cell contacts (Kharfallah et al., 2017). These have been considered to represent active PCP signaling sites. In untransduced MDCK cells, endogenous OTULIN was distributed in puncta throughout the cytoplasm (Fig. 3A). However, in the presence of either membrane-localized GFP-VANGL1 or GFP-VANGL2, OTULIN showed membrane or juxtamembrane distribution, as detected by immunofluorescence with an anti-OTULIN antibody (Fig. 3A-F). We quantified the localization of OTULIN and GFP-VANGL1 or GFP-VANGL2 to either ‘free’ edges of cells (i.e. those that are not in contact with a neighboring cell) or edges that are in contact with neighboring cells (Fig. 3G). The ‘free’ edges of cells showed OTULIN juxtamembrane localization in only 7% and 12% of untransduced and GFP-VANGL1-transduced cells, respectively, and in 17% of GFP-VANGL2 transduced cells. GFP-VANGL1 and GFP-VANGL2 were localized to 33% and 36% of free edges in respective cell lines. Although present only at 3% of cell-cell contacts in untransduced cells, OTULIN could be detected at 73% and 78% cell-cell contacting edges of GFP-VANGL1 and GFP-VANGL2 transduced cells, respectively, and GFP-VANGL1 and GFP-VANGL2 themselves were found at 96% and 92% of cell-cell contacts. Additionally, when present on free edges, the expression of OTULIN and GFP-VANGL1/2 appeared more diffuse, and not restricted to a defined boundary near the membrane. As in HEK293 cells, endogenous HOIP was distributed throughout the cytoplasm of all MDCK cell lines with no obvious enrichment near cell membranes (Fig. S2). Thus, just as VANGL1/2 proteins have been shown to recruit SCRIB in MDCK cells (Kallay et al., 2006) and Dvl2 in *Xenopus* during convergent extension (Seo et al., 2017), VANGL1/2-containing PCP complexes can recruit endogenous OTULIN to the plasma membrane at sites of cell-cell contacts, i.e. sites of presumptive active PCP signaling.

### Both SCRIB and VANGL2 associate with Met1-Ub conjugated proteins, but only VANGL2 is modified with Met1-Ub

**Fig. 4.**
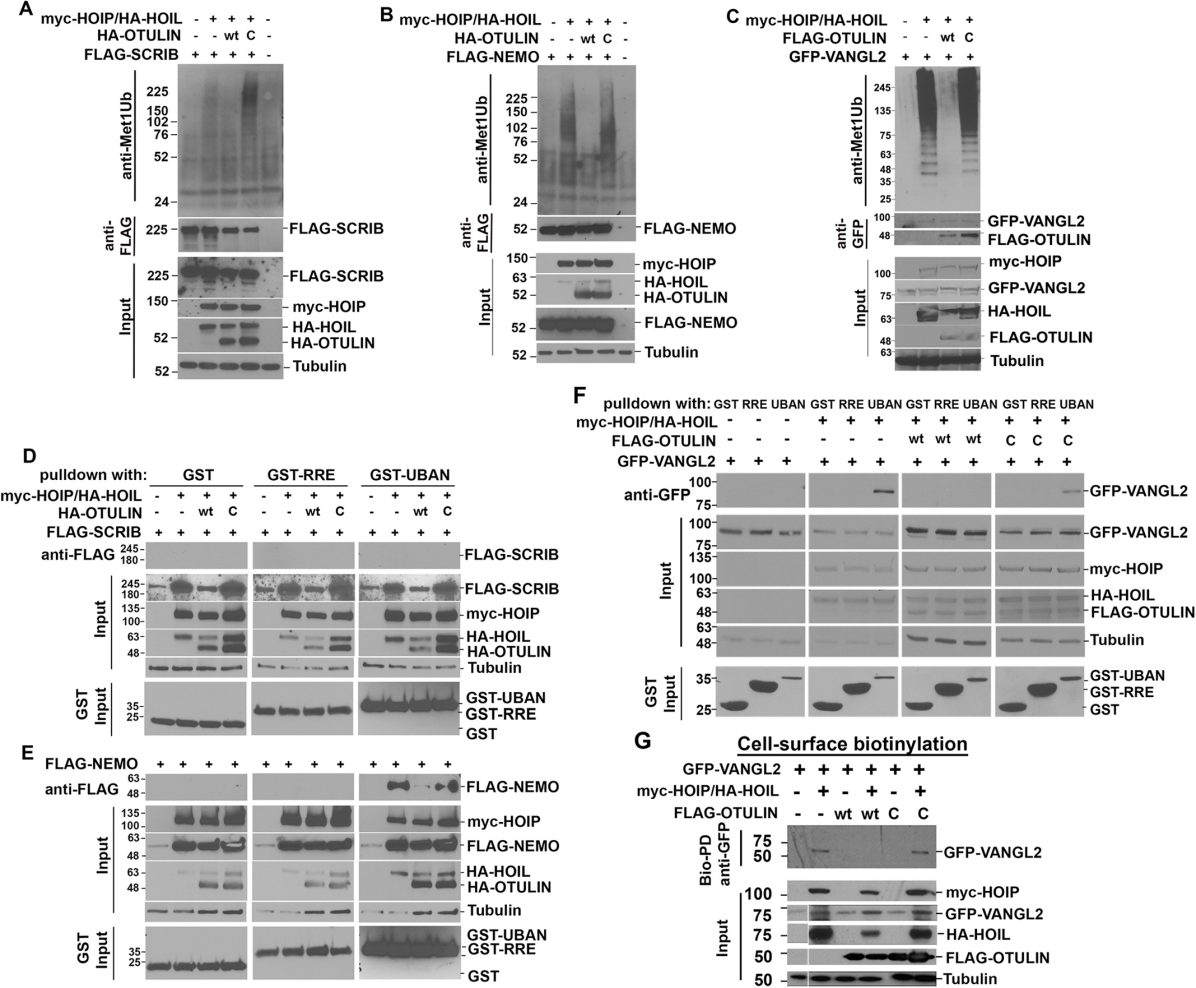
**VANGL2, but not SCRIB, is modified with Met1-Ub chains, and linear ubiquitination correlates with VANGL2 surface presentation.** (A-C) A Met1-Ub antibody detected proteins modified with Met1-Ub chains in immunoprecipitates of (A) FLAG-SCRIB and (B) FLAG-NEMO recovered with an anti-FLAG antibody, and (C) GFP-VANGL2 recovered with an anti-GFP antibody from HEK293T cells transfected with HA-HOIL and myc-HOIP. Adding HA-OTULIN (‘wt’), but not HA-OTULIN^C129S^ (‘C’), eliminated Met1-Ub immunoreactivity. Input amounts for each construct and tubulin levels are shown. (D-F) Purification of Met1-Ub-conjugated (D) FLAG-SCRIB, (E) FLAG-NEMO and (F) GFP-VANGL2 using GST protein, GST-coupled to the Met1-Ub-binding UBAN domain of NEMO (GST-UBAN) or to a mutated non-Met1-Ub-binding UBAN domain (GST-RRE). Pull-down of GST-UBAN recovered (E) FLAG-NEMO and (F) GFP-VANGL2, but not (D) FLAG-SCRIB from HA-HOIL/myc-HOIP-expressing HEK293 cells. The addition of HA-OTULIN (‘wt’), but not catalytically inactive HA-OTULIN^C129S^ (‘C’), abrogated recovery of FLAG-NEMO and GFP-VANGL2. (G) A cell surface biotinylation assay recovered biotinylated GFP-VANGL2 from HEK293T cells transfected with HA-HOIL and myc-HOIP, as shown in a representative immunoblot probed with anti-GFP antibody; ‘Bio-PD’ indicates biotin pulldown. The presence of FLAG-OTULIN (‘wt’), but not FLAG-OTULIN^C129S^ (‘C’), eliminated surface biotinylation of GFP-VANGL2. Immunoblots are representative of three independent experiments.

These observations suggested that SCRIB and/or associated PCP complex proteins might be modified with Met1-Ub. Consistent with this expectation, an anti-Met1-Ub antibody detected a diffuse immunoreactive signal in immunoprecipitates of FLAG-SCRIB, GFP-VANGL2 and FLAG-tagged NEMO (also called IKK-γ or IKBKG; a known LUBAC/OTULIN client; Niu et al., 2011) when they were co-expressed with HA-HOIL and myc-HOIP. Moreover, adding HA-OTULIN eliminated Met1-Ub immunoreactivity, but co-transfection with catalytically inactive HA-OTULIN^C129S^ reproducibly increased this signal (Fig. 4A-C). Similarly, immunoprecipitation of GFP-PRICKLE1 also recovered Met1-Ub modified proteins (Fig. S3).

Next, we tested whether Met1-Ub chains might be conjugated to SCRIB or VANGL2. Pulldowns using the Met1-Ub-binding domain (UBAN) from NEMO (Fiil et al., 2013; Rodgers et al., 2014; Takiuchi et al., 2014) fused to glutathione S-transferase (GST) could recover Met1-Ub-modified proteins and recovered FLAG-NEMO, GFP-VANGL2 and GFP-PRICKLE1, but not FLAG-SCRIB from myc-HOIP/HA-HOIL-co-expressing HEK293 cells (Fig. 4D-F; Fig. S3B). GST alone or GST fused to a mutated non-Met1-Ub-binding UBAN domain (GST-RRE) did not recover any of these proteins (Fiil et al., 2013; Rodgers et al., 2014) (Fig. 4D-F). The presence of OTULIN, but not HA-OTULIN^C129S^, abrogated the recovery of FLAG-NEMO (Fig. 4E) and GFP-VANGL2 by GST-UBAN (Fig. 4F). Thus, Met1-Ub chains are likely attached to VANGL2 and PRICKLE1 but not SCRIB.

### LUBAC and OTULIN regulate the surface presentation of VANGL2

Because SCRIB is required for VANGL2 trafficking, we asked whether LUBAC and OTULIN might regulate the cell surface presentation of VANGL2. Using a surface biotinylation assay, we found that biotinylated GFP-VANGL2 was recovered from HEK293T cells co-transfected with myc-HOIP and HA-HOIL (Fig. 4G). Co-transfection of FLAG-OTULIN, but not FLAG-OTULIN^C129S^, abrogated GFP-VANGL2 surface presentation. Thus, VANGL2 surface presentation correlates with its linear ubiquitination status.

### Neural tube malformations in *Otulin^Δex3/Δex3^* embryos

**Fig. 5.**
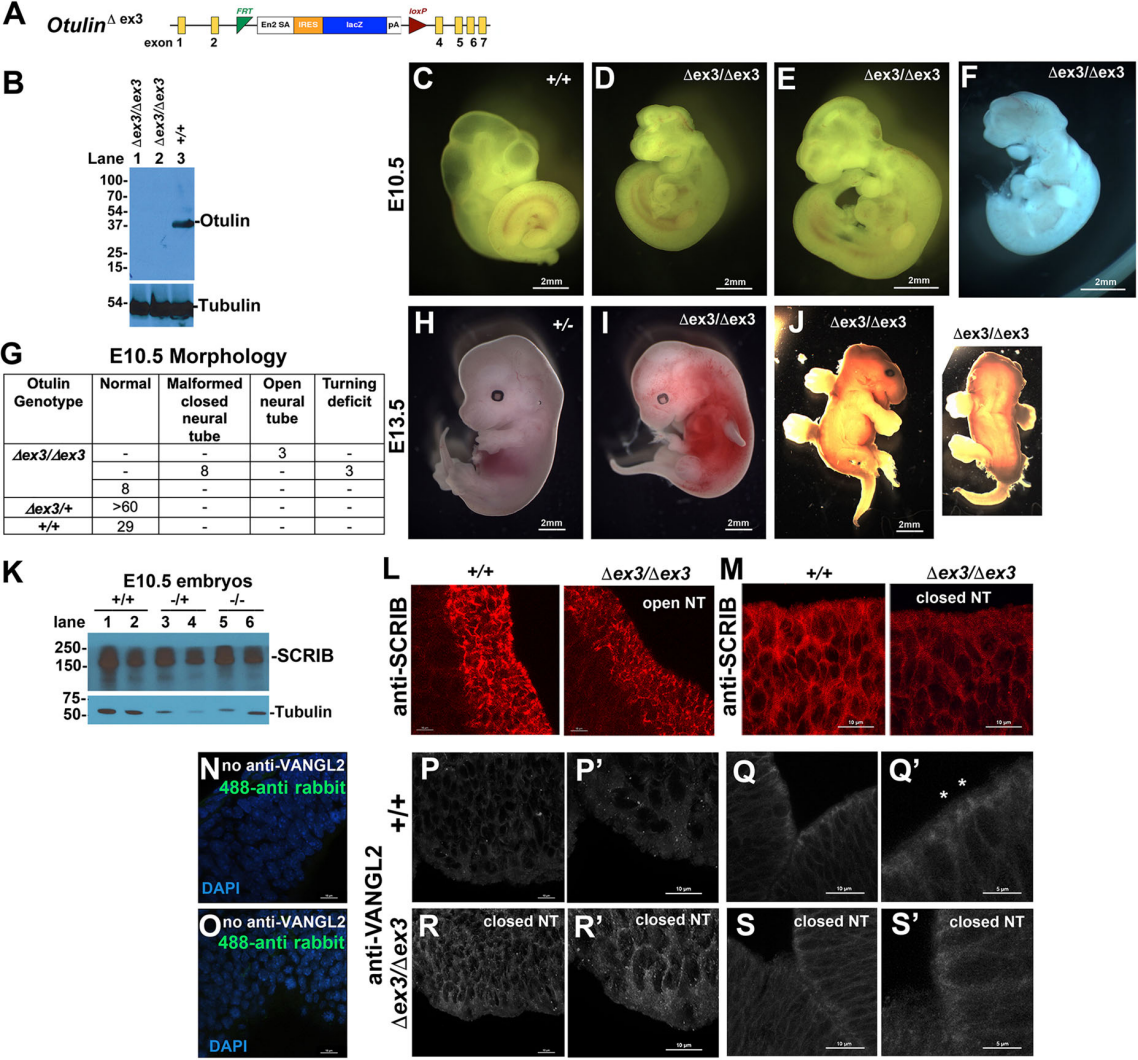
**Mice homozygous for the *Otulin^Δex3^* null allele show variable neural tube deficits and VANGL2 localization.** (A) Schematic diagram of the *Otulin^Δex3^* allele. (B) An immunoblot using a polyclonal anti-OTULIN antibody detected the 34 kDa OTULIN protein in *Otulin^+/+^* E10.5 embryos, but not in their *Otulin^Δex3/Δex3^* littermates. (C-F) At E10.5, *Otulin^Δex3/Δex3^* embryos (D-F) showed collapsed hindbrain roofplates and variable head morphologies relative to a representative *Otulin^+/+^* littermate (C). (G) The table summarizes the number of *Otulin^Δex3^* allele-carrying embryos exhibiting neural tube malformations or laterality turning deficits at E10.5. Resorbing embryos were excluded from scoring. (H-J) At E13.5, relative to *Otulin^Δex3/+^* littermates (H), *Otulin^Δex3/Δex3^* embryos (I,J) appeared smaller, exhibited curly tails, widespread hemorrhages (I) and sometimes an open neural tube (J). (K) SCRIB protein levels in E10.5 *Otulin^Δex3/+^* and *Otulin^Δex3/Δex3^* embryonic lysates, as detected in an immunoblots probed with anti-SCRIB and anti-tubulin antibodies. (L,M) Immunofluorescence using an anti-SCRIB antibody detected SCRIB localized to cell boundaries in neural tubes of E10.5 *Otulin^Δex3/Δex3^* embryos with open (L) and closed (M) neural tubes. (N-S′) VANGL2 localization in hindbrains of *Otulin^+/+^* (N-Q′) and *Otulin^Δex3/Δex3^* (R-S′) embryos at E10.5. (N,O) Secondary antibody only control. (P-S′) Anti-VANGL2 staining in *Otulin^Δex3/Δex3^* embryos (R-S′) with closed neural tubes appeared similar to that in WT littermates (P-Q′), except for more punctate appearance in some mutant embryos or occasional subtle differences in localized concentration of VANGL2 in cells facing the hindbrain ventricle (asterisks in Q′). P′-S’ show higher magnification views of P-S. Images are representative of three or more sections from six embryos per genotype. Scale bars: 2 mm (C-F,H-J); 10 µm (L-S,P′,R′); 5 µm (Q′,S′).

Previously identified as causative of *gumby* mutant mouse phenotypes (Rivkin et al., 2013), the *Otulin^W96R^* and *Otulin^D336E^* mutations (Rivkin et al., 2013) and the CRISPR-generated *Otulin^C129A^* mutation (Heger et al., 2018) all compromise OTULIN catalytic activity but not its PBM motif and do not impact overall OTULIN levels. Thus, to examine the consequences of OTULIN loss, we generated *Otulin* knockout mice, in which excision of exon 3 introduces a premature stop codon predicted to result in either a truncated OTULIN protein or, owing to nonsense-mediated decay, little or no protein (Fig. 5A,B). Accordingly, in immunoblots of lysates from *Otulin^Δex3/Δex3^* embryos, the WT protein (34 kDa) or predicted truncated proteins were not detected (Fig. 5B). At embryonic day (E) 10.5, the roofplates of *Otulin^Δex3/Δex3^* embryos tended to appear collapsed, and often their heads were reduced and variably misshapen (Fig. 5C-F). As previously reported for *Otulin^W96R/W96R^* and *Otulin^D336E/D336E^* mutants, *Otulin^Δex3/Δex3^* embryos appeared unusually vascularized, had branchial arch defects and died between E11.5 and 13.5 (Fig. 5H-J). One of the rare *Otulin^Δex3/Δex3^* embryos that survived to E13.5 (Fig. 5J) and three out of 38 E10.5 *Otulin^Δex3/Δex3^* embryos exhibited two additional phenotypes reminiscent of *Vangl2* and PCP mouse mutants: mildly curly tails and completely open neural tubes. Moreover, VANGL2 has been implicated in left-right patterning, which is established around E8.5 and directs embryo turning*.* Three out of 38 homozygous embryos exhibited an embryonic turning phenotype, absent in 41 WT and over 60 heterozygous embryos examined (Fig. 5G).

Next, we examined SCRIB and VANGL2 in the hindbrains of *Otulin^Δex3/Δex3^* embryos. *Otulin* loss did not affect SCRIB protein levels in E10.5 *Otulin^Δex3/+^* and *Otulin^Δex3/Δex3^* embryos (Fig. 5K). Immunofluorescence using an anti-SCRIB antibody detected SCRIB localized to cell boundaries in the neural tubes of E10.5 WT and *Otulin^Δex3/Δex3^* embryos. Although the SCRIB protein showed some slightly more concentrated regions in cells lining the hindbrain ventricle in WT relative to *Otulin^Δex3/Δex3^* embryos with open neural tubes (Fig. 5L), WT and *Otulin^Δex3/Δex3^* embryos with closed neural tubes did not show marked differences in SCRIB distribution (Fig. 5M). VANGL2 was enriched at the membranes of cells lining the hindbrain ventricles of *Otulin^+/+^* (Fig. 5P-Q′) and *Otulin^Δex3/Δex3^* embryos with closed neural tubes (Fig. 5R-S′) and outlined cell membranes throughout the hindbrain. No staining was observed in the negative controls (Fig. 5N,O). Some WT littermates sometimes showed brighter regions of VANGL2 accumulation at cell surfaces facing the ventricles (marked with asterisks in Fig. 5Q′), which were not as apparent in *Otulin^Δex3/Δex3^* embryos (Fig. 5S,S′). In some *Otulin^Δex3/Δex3^* embryos, slightly brighter overall VANGL2 immunofluorescence signal could be seen. These differences were both subtle and – although consistent within a given embryo – variable between mutant embryos. Moreover, VANGL2 distribution could not be used to reliably identify mutant embryos by observers who were not aware of the genotypes. Thus, although the effects of OTULIN loss on neural tube morphology could be due to a subtle, highly dynamic role of Met1-Ub on VANGL2 trafficking, they might also be secondary to disrupted angiogenesis and impending embryonic lethality. Taken together with the observation that other disruptions of VANGL2 trafficking, such as those seen upon Sec24b loss (Merte et al., 2010), are also difficult to assess in the mouse neural tube, we chose to test the impact of OTULIN loss on PCP signaling further in another *in vivo* system.

### OTULIN loss disrupts Wnt5a responses in MDA-MB-231 cells

**Fig. 6.**
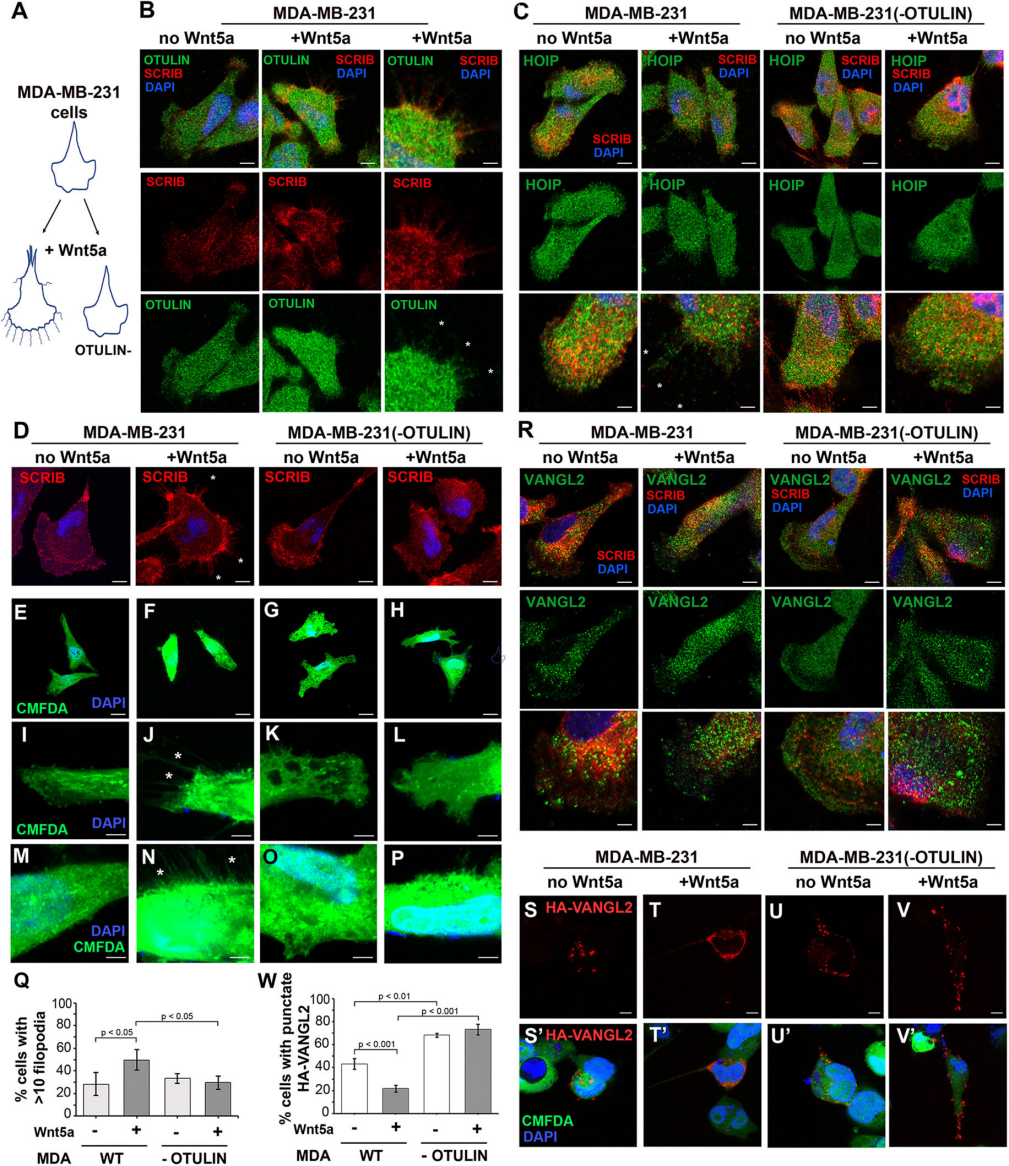
**OTULIN loss disrupts Wnt5a responses in MDA-MB-231 cells.** (A) Schematic diagrams depict Wnt5a-induced filopodia extension in MDA-MB-231 cells with and without OTULIN. (B,C) Immunofluorescence analyses reveal (B) OTULIN (green) and (C) HOIP (green) presence throughout MDA-MB-231 cells and along with SCRIB (red) in processes upon Wnt5a treatment. Righthand panels in B and bottom panels in C show higher-magnification views. (D) Immunofluorescence analyses with a rabbit anti-SCRIB antibody show SCRIB (red) localization in cells with or without OTULIN. (E-P) Impact of OTULIN loss on Wnt5a-dependent filopodia formation in MDA-MB-231 cells, as detected with green Cell Tracker Green CMFDA. In contrast to control medium-treated MDA-MB-231 cells (E,I,M), Wnt5a-stimulated MDA-MB-231 cells (F,J,N) extended many fine filopodia. Neither unstimulated (G,K,O) nor Wnt5a-stimulated (H,L,P) MDA-MB-231 (−OTULIN) cells showed increased filopodia extension. Asterisks (B-D,J,N) mark examples of filopodia. (Q) Quantification of the percentage of cells with more than ten filopodia. Data were analyzed using Student’s one-tailed paired *t*-test, type 2 (two-samples; assuming equal variance). Error bars show standard deviation of error. (R) VANGL2 (green) was broadly expressed in MDA-MB-231 cells regardless of OTULIN presence and was present in Wnt5a-induced processes in MDA-MB-231 cells with OTULIN. SCRIB immunofluorescence is shown in red. (S,S′) Upon treatment with control medium, transfected HA-VANGL2 (red) was present in a few puncta in the cytoplasm. (T,T′) Upon treatment with Wnt5a, HA-VANGL2 was distributed in the cytoplasm and towards the membrane. (U-V′) MDA-MB-231 cells lacking OTULIN showed punctate cytoplasmic distribution in unstimulated (U,U′) and Wnt5a-stimulated (V,V′) cells. Cells were incubated with CellTracker Green CMFDA (green) and immunostained for HA-VANGL2 (red), and the nucleus was stained with DAPI. (W) Quantification of percentage of cells with punctate VANGL2 localization for unstimulated and Wnt5-stimulated MDA-MB-231 and MDA-MB-231 (−OTULIN) cells. Images are representative of three or more independent experiments. Scale bars: 10 µm (B, left and middle columns; C, top and middle rows; D,S-V); 5 µm (B, right column; C, bottom row; I-P,R); 20 µm (E-H).

Because MDA-MB-231 breast cancer cells are an established cell culture system used to study responses to PCP ligands, such as Wnt5a, we examined the consequences of *OTULIN* loss on Wnt5a responses in these cells (Fig. 6A). We found that MDA-MB-231 cells are aneuploid for the *OTULIN*-harboring region on chromosome 5 and, using CRISPR-Cas9, generated MDA-MB-231 *OTULIN*^−/−^ cells, which did not express detectable OTULIN (Fig. S4). Lack of suitable reagents prevented us from testing whether endogenous SCRIB and OTULIN could be co-immunoprecipitated with each other under unstimulated or Wnt5a-treated conditions. However, immunofluorescence analyses revealed that endogenous OTULIN was expressed throughout MDA-MB-231 cells and, upon Wnt stimulation, could also be seen in fine SCRIB-expressing processes extending from the leading edge (Fig. 6B). SCRIB, HOIP and VANGL2 could also be detected in these Wnt5a-induced processes (Fig. 6C,D,R). OTULIN loss did not detectably impact cytoplasmic localization of SCRIB, VANGL2 or HOIP in untreated or Wnt5a-treated cells (Fig. 6C,D,R) but, as reported for some other cell types (Fiil et al., 2013), reduced overall levels of HOIP (Fig. S4). Interestingly, far fewer MDA-MB-231 *OTULIN*^−/−^ cells extended processes in response to a 4 h Wnt5a stimulation (Fig. 6E-Q). We used CellTracker Green (5-chloromethylfluorescein diacetate) CMFDA staining to visualize these processes better and found that Wnt5a stimulation doubled the number of MDA-MB-231 cells extending an abundance of fine processes (51.0±9.1%, indicated as mean±s.d.) relative to unstimulated conditions (28.0±10.2%). By contrast, there was no difference in the percentage of MDA-MB-231 *OTULIN*^−/−^ cells extending fine processes under unstimulated (33.0±4.2%) or Wnt5a-treated (33.0±4.2%) conditions.

Because MDA-MB-231 *OTULIN*^−/−^ cells have deficits in process extension and because VANGL2 is abundant throughout the cytoplasm (Fig. 6R), effects of OTULIN loss on Wnt5a-dependent VANGL2 trafficking in general or localization to processes specifically could not be assessed adequately. However, previous studies have found that Wnt5a induces readily trackable redistribution of transfected HA-VANGL2 in MDA-MB-231 cells (Luga et al., 2012). As previously reported, we observed that upon treatment with control medium, HA-VANGL2 was localized in puncta within 43.3±7.4% of MDA-MB-231 cells (Fig. 6S,S′). Wnt5a-conditioned medium induced HA-VANGL2 redistribution throughout the cytoplasm and towards the membrane so that only 21.7±4.8% of stimulated WT MDA-MB-231 cells, i.e. approximately half as many treated versus untreated WT cells, still contained HA-VANGL2 puncta (Fig. 6T,T′). Strikingly in Wnt5a-stimulated MDA-MB-231 *OTULIN*^−/−^ cells, HA-VANGL2 remained in cytosolic puncta in 73.3±7.7% of cells, i.e. to the same degree as in unstimulated mutant cells (68.3±2.6%) (Fig. 6U-W). Thus, OTULIN is required for Wnt5a-induced filopodia extension and HA-VANGL2 trafficking in this PCP model system.

## DISCUSSION

Here, we have shown that OTULIN, via its PBM, can interact with SCRIB. Underscoring the remarkable selectivity of OTULIN PBM-dependent interactions, out of the >300 PDZ domain-containing proteins (reviewed in Lee and Zheng, 2010), only three, including SCRIB, showed high-confidence interactions with OTULIN in our study. Even across all studies reported in the BioGRID database (Oughtred et al., 2021), only the PDZ domain-containing proteins SYNJ2BP and PDZK1 (NHERF3) were additionally reported as human OTULIN interactors – all from the BioPlex dataset (Huttlin et al., 2021). The OTULIN-SNX27 interaction requires the OTULIN PBM and contacts between additional residues within the OTU domain (Stangl et al., 2019). In AP-MS – although not in immunoprecipitation – experiments, loss of the OTULIN N-terminus reduces OTULIN-SCRIB, but not OTULIN-SNX27, interactions. Thus, an emerging theme is that, aside from the PBM itself, additional OTULIN sequences and possibly protein partners help secure it to PDZ domain-containing protein complexes.

Upon uncovering the OTULIN-SCRIB interaction and evidence that Met1-Ub modification of VANGL2 promoted its surface presentation in HEK293 cells, we probed established PCP hallmarks in several PCP model systems. In canine MDCK cells, in which VANGL1/2 clustering at cell-cell contacts has been regarded as an indicator of the formation of activated, functional PCP complexes, we found that endogenous OTULIN was enriched at these sites. MDA-MB-231 cells are an established model with which to examine immediate responses to Wnt5a signaling. In these cells, OTULIN and HOIP were found in Wnt5a-induced processes. OTULIN loss compromised both Wnt5a-induced process extension and Wnt5a induced redistribution of transfected HA-VANGL2. Taken together, these observations support a role of OTULIN in PCP signaling.

The OTULIN-SCRIB interaction is likely cell type- and condition-specific. It was not recovered in AP-MS experiments performed on Jurkat T lymphocyte cells, which express both SCRIB (Petit et al., 2005) and HOIP (Stangl et al., 2019) endogenously. Moreover, as in other cases, activation of signaling might be required for the OTULIN-SCRIB association. For instance, OTULIN is only recruited to TNF receptor 1 (TNFR1 or TNFRSF1A) (Schaeffer et al., 2014; Takiuchi et al., 2014; Draber et al., 2015; Elliott et al., 2016) or NOD2 (Fiil et al., 2013) signaling complexes upon activated signaling. In a similar manner, we observed co-localization of endogenous OTULIN with presumptive activated GFP-VANGL1/2-containing PCP complexes at cell-cell contacts in MDCK cells.

In vertebrates, PCP is likely achieved in a step-wise manner: First, VANGL proteins bind Dvls (Park and Moon, 2002; Bastock et al., 2003; Torban et al., 2004b; Suriben et al., 2009) and Pk (Jenny et al., 2003) and recruit these to the membrane. Subsequently, cellular asymmetry of core PCP proteins is achieved through the mutual antagonism of SCRIB-VANGL2-Pk and Dvl-Fzd receptor complexes, which drives both their rapid turnover at the plasma membrane and stabilization within opposing domains (Strutt et al., 2011). Ultimately, the VANGL/Pk complex is partitioned to locations separate from those of Dvls (Bastock et al., 2003; Chen et al., 2008; Vladar et al., 2009). The reciprocal sensitivity to proteasomal inhibition, which stabilizes FLAG-OTULIN interactions with HA-Dvl2 (Rivkin et al., 2013) but reduces FLAG-OTULIN interactions with endogenous SCRIB or HOIP in AP-MS, would be consistent with involvement in such reciprocal subcellular partitioning.

The Wnt pathway proteins VANGL2 and PRICKLE1 join the limited number of receptor-associated proteins, which include RIPK1, RIPK2, TRADD, MYD88, IRAK1, IRAK4 and NEMO (Tokunaga et al., 2009; Gerlach et al., 2011; Emmerich et al., 2013; Fiil et al., 2013; Draber et al., 2015; Hrdinka et al., 2016), known to be modified with Met1-Ub chains. Ubiquitin-dependent processes and particularly degradative ones have been shown to refine the subcellular localization of PCP components. For instance, the C2-WW-HECT-type ubiquitin ligases Smad ubiquitination regulatory factors 1 (Smurf1) and 2 (Smurf2) are recruited to sites of activated Dvl2, where they ubiquitinate Pk and target it for degradation – an event expected to reduce local VANGL1/2 surface presentation, direct cellular orientation and promote PCP signaling (Narimatsu et al., 2009). Conversely, in cultured breast cancer cells, VANGL proteins act as a scaffold for the ring finger-containing E3 ligase neuregulin receptor degradation protein 1 (Nrdp1; also called RNF41, FLRF or RBCC), which promotes the K63-linked polyubiquitination of the DEP domain of the Dvl proteins, thereby blocking Dvl binding to the plasma membrane (Wald et al., 2017). Wnt signaling can also be limited by a feedback loop that relies on the endocytosis of Fzd receptors ubiquitinated by the E3 ligases RNF43 and ZNRF3, which themselves are upregulated upon Wnt signaling (Hao et al., 2012; Koo et al., 2012). Met1-Ub stands apart from these ubiquitin-based events by increasing the surface presentation of the VANGL2 protein.

The linear ubiquitination status adds a chordate-specific layer to the regulation of SCRIB-dependent trafficking of VANGL2. The OTULIN interactors SNX27 and SCRIB (Belotti et al., 2013) have both been shown to promote VANGL2 trafficking to the plasma membrane. Although different components of the protein sorting machinery, such as the GTP-binding protein Arfrp1, the clathrin adaptor complex 1 (AP-1) (Guo et al., 2013; Carvajal-Gonzalez et al., 2015) and the coat protein complex II (COPII) component Sec24b (Mancias and Goldberg, 2007, 2008; Bonnon et al., 2010), act with remarkable selectivity in the stepwise targeting of VANGL2, none of these were recovered as OTULIN interactors. By associating with the retromer, SNX27 promotes the recycling of transmembrane proteins from endosomes to the plasma membrane. Accordingly, *Snx27^−/−^* mouse embryonic fibroblasts show reduced VANGL2 protein levels and cell surface presentation (Wang et al., 2016). Supported by the observation that both catalytically active and inactive OTULIN can inhibit membrane presentation of glucose transporter 1 (GLUT1) and trafficking of a GFP-SLC1A4 reporter, the OTULIN-SNX27 interaction has been proposed to block the VPS26A-dependent association of SNX27 with the retromer (Stangl et al., 2019). However, only catalytically active OTULIN can inhibit surface presentation of VANGL2. Thus, the OTULIN-SCRIB interaction and linear (de)ubiquitination are likely to play the determining role in Wnt5a-responsive and OTULIN-dependent VANGL2 trafficking.

*Otulin^Δex3/Δex3^* mice can show a range of neural tube malformations, which may be a direct consequence of disrupted PCP or secondary to other deficits, e.g. impending embryonic lethality owing to angiogenic malfunction and inflammation. Rare *Otulin^Δex3/Δex3^* embryos exhibit an open neural tube, as is also seen in mice with mutations in *Scrib* (Montcouquiol et al., 2003; Murdoch et al., 2003; Stottmann et al., 2011), *Vangl2* (Strong and Hollander, 1949), *Ptk7* (Paudyal et al., 2010), the CELSR mutants *spin cycle* and *crash* (Curtin et al., 2003), and ones carrying combined mutations of *Dvl1*/*Dvl2* or *Fz3*/*Fz6* (Wang et al., 2006). That said, as for *Otulin^Δex3/Δex3^* embryos, the severity of neural tube deficits seen in many PCP component mutants can be highly variable. For example, only 2-3% of *Dvl2*^−/−^ mice show obvious neural tube deficits – presumptively owing to redundancy with *Dvl1* (Hamblet et al., 2002). Notably, VANGL2 deficiency-mediated PCP deficits are genetically and likely environmentally sensitive, as the tail loops or kinks are seen in only 11% of *Vangl2^ΔTMs/+^* mice on a C57BL6 genetic background and are absent altogether on FVB or A/J inbred backgrounds (Yin et al., 2012). As is the case for the effects of OTULIN on VANGL2 localization in mouse embryos, only mild effects have been reported for mouse E9.5 embryonic cardiomyocytes (Phillips et al., 2007) and E14.5 lung epithelia (Yates et al., 2013) lacking *Scrib*, and in the neuroepithelium of *Sec24b* mutants (Merte et al., 2010), in which VANGL2 distribution appears at best mildly more concentrated within the cytosol relative to its concentration at apical cell membranes. However, as reported for disease-associated variants in core PCP components, manipulation of OTULIN activity or levels disrupts VANGL2 protein membrane trafficking consistently and strikingly in cell culture (Kharfallah et al., 2017).

Inflammation can induce Wnt5a and PCP signaling, which in turn can amplify the production of pro-inflammatory cytokines (reviewed in Pashirzad et al., 2017). TNFR1 loss suppresses *Otulin*, *Hoil* and *Hoip* embryonic phenotypes in mice (Peltzer et al., 2014, 2018; Heger et al., 2018). Limiting TNF-based pro-inflammatory responses also improves symptoms in patients homozygous for rare variants in *OTULIN* and suffering from an autoinflammatory syndrome called OTULIN-related autoinflammatory syndrome (Damgaard et al., 2016) or otulipenia (Zhou et al., 2016). Of relevance to developmental PCP signaling, at the onset of scoliosis in zebrafish lacking the vertebrate-specific PCP component Protein tyrosine kinase 7 (Ptk7), TNF is highly expressed and triggers Il-6 expression (Van Gennip et al., 2018). Notably, treatment with non-steroidal anti-inflammatory drugs – depending on the timing of administration – halts scoliosis progression or prevents it altogether (Hayes et al., 2014). Our observations suggest that it is worth considering the possibility that reduced levels of *OTULIN* might contribute to the scoliosis reported in 30 of the surveyed 73 cri du chat syndrome patients, who are haploinsufficient for the *OTULIN*-harboring region on chromosome 5p (Honjo et al., 2018).

Finally, the presence of Met1-Ub on VANGL2/Pk-containing PCP complexes makes it attractive to speculate that Met1-Ub-binding domain-containing proteins may stabilize and concentrate PCP complexes in specific cellular locales or integrate PCP signaling with pro-inflammatory NFκB signaling pathways (reviewed in Dikic et al., 2009; Hrdinka and Gyrd-Hansen, 2017; Fennell et al., 2018). NEMO, optineurin (OPTN) and the three A20-binding inhibitors of NFκB (ABIN1-3 or TNIP1-3) harbor structurally related UBAN domains, which, by forming homodimers, permit simultaneous binding of two linear chains (Rahighi et al., 2009; Nakazawa et al., 2016; Hauenstein et al., 2017; Li et al., 2018). Thus, these or other Met1-Ub-binding proteins, such as A20 (also called TNFAIP3) (Tokunaga et al., 2012), might expand VANGL-Pk-containing PCP signaling hubs and/or integrate PCP complexes with other Met1-Ub modified receptors, such as TNFR1.

### Limitations and open questions

The interaction between OTULIN and SCRIB offers the first critical step for dynamic OTULIN-promoted process extension in Wnt5a-stimulated MDA-MB-231 cells. The precise steps following OTULIN recruitment to PCP complexes and leading to Met1-Ub addition and removal to PCP complexes to regulate VANGL2 membrane presentation remain to be elucidated. In the systems examined here, the subcellular distribution of endogenous HOIP and Met1-Ub is broad and unaffected by OTULIN overexpression or loss of Wnt5 stimulation. This and the unavailability of suitable reagents has limited further mechanistic tests in this study.

Other biologically relevant Wnt5a-dependent processes, for example, cell-specific responses to infection (Schaale et al., 2011; Chen et al., 2017; Jati et al., 2020) or SCRIB-dependent processes – including ones directing apical-basal polarity – may be impacted by the OTULIN-SCRIB interaction and Met1-Ub homeostasis. Given the likely dynamic role(s) of the OTULIN-SCRIB interaction, these might be best explored in systems that can be facilely manipulated *in vivo* and *ex vivo*.

The importance of Met1-Ub and OTULIN in VANGL2 trafficking and Wnt5a-dependent responses sets the stage for exploring exciting mechanistic and biological questions. Does OTULIN reside at PCP complexes in an active or inactive state? How is OTULIN activity or localization at SCRIB-containing complexes mediated? As *in vitro*, a phosphomimetic mutant of the PBM sequence sharing the four C-terminal amino acids (ETSL) with the OTULIN PBM showed enhanced interactions with SCRIB PDZ domains 2 and 3 (Sundell et al., 2018), kinases and phosphatases are likely candidates for disrupting or priming the OTULIN-SCRIB interaction. It is unclear whether Met1-Ub-modified PCP complexes are interlinked with immune or inflammatory Met1-Ub-modified signaling complexes. The findings presented here offer a launching pad for further important advances in the understanding of vertebrate PCP, of human conditions such as cri du chat syndrome, and possibly also the links between PCP-dependent tissue remodeling and inflammation.

## MATERIALS AND METHODS

### Mouse husbandry

All mouse husbandry and handling were performed in conformity with the Canadian Council of Animal Care recommendations (AUP 0024a-00H). Mice carrying the conditional *Otulin* allele, in which exon 3 is flanked by LoxP sites, were generated by the Toronto Centre of Phenogenomics from two C57BL/6N embryonic stem cell lines (Fam105b clones HEPD0595_8_G07 and HEPD0595_8_C10) obtained from EUCOMM. Both embryonic stem cell lines produced mice that, when homozygous for the *Otulin* mutant allele, showed the expected embryonic lethality and angiogenic deficits. Crossing these mice with ones carrying the widely expressed B6.C-Tg (CMV-Cre)1Cgn/J transgene generated *Otulin^Δex3/+^* mice, in which exon 3 is deleted, as determined by PCR-based analyses. Deletion of exon 3 introduces a premature stop codon that truncates the OTULIN protein at amino acid 76 (*Otulin^1-76^*), and RT-PCR analyses and sequencing confirmed the consequences on *Otulin* mRNA.

### Cell culture for mass spectrometry

The plasmids used in this study are listed in Table S3. Most OTULIN expression constructs were previously described in Rivkin et al. (2013). N-terminal truncation constructs (i.e. OTULIN**^Δ^**^54^) were cloned into FLAG-pcDNA3.1 [Network Biology Collaborative Centre (NBCC), Lunenfeld-Tanenbaum Research Institute (LTRI)] and full-length OTULIN and OTULIN^C105X^ constructs were cloned into FLAG-pcDNA5-FRT/TO (NBCC, LTRI). HEK293 (Δ54 cell lines) or HEK293 Flp-In/T-REx (WT cell lines) cells were transfected with FLAG-tagged OTULIN expression constructs using Effectene (QIAGEN) in six-well plates. Cells from each transfection were split to 10 cm plates one day after transfection at three serial dilutions (pool A, 1:10; pool B, 1:100; pool C,1:250), selected with G418 (Millipore) or hygromycin B (Invitrogen) and maintained until the formation of colonies was observed. Once colonies were obtained, the selected plates were pooled and expanded for AP-MS experiments.

FLAG-OTULIN and empty FLAG-vector pool A cells were cultured in Hyclone Dulbecco's modified Eagle medium (DMEM; Thermo Fisher Scientific), supplemented with 5% fetal bovine serum (FBS; Invitrogen), 5% cosmic calf serum (Thermo Fisher Scientific), and 100 U/ml penicillin/streptomycin (Thermo Fisher Scientific). At 60-70% confluence, cells were induced with 1 µg/ml tetracycline for 24 or 48 h. After protein induction, cells were harvested by scraping with a rubber cake spatula, washed once with PBS and pelleted by centrifugation (500 ***g***, 5 min, 4°C). Cell pellets were frozen on dry ice and stored at −80°C until needed.

### AP-MS

Cell pellets were lysed on ice by adding a 1:4 (pellet weight:volume) ratio of cold lysis buffer [50 mM HEPES-KOH pH 8.0, 100 mM KCl, 2 mM EDTA, 0.1% NP40, 10% glycerol, 1 mM PMSF, 1 mM DTT and protease inhibitor cocktail (Sigma-Aldrich; P8340; 1:500)]. After lysis, cells were subjected to one freeze-thaw cycle (on dry ice) and the lysates were cleared by centrifugation (20,800 ***g***, 20 min, 4°C). AP was performed by incubating cleared lysates with 12.5 µl bed volume of pre-washed anti-FLAG M2 magnetic beads (Sigma-Aldrich; M8823) for 2 h. After affinity purification, cell lysates were aspirated and beads washed once with lysis buffer (without 1 mM DTT, 1 mM PMSF, or protease inhibitor cocktail at 1:500), followed by one wash with 20 mM Tris-HCl (pH 8.0) containing 2 mM CaCl_2_, before bound proteins were subject to on-bead digestion for 4 h with 500 ng trypsin (Sigma-Aldrich; T6567) in 20 mM Tris-HCl (pH 8.0) at 37°C. After 4 h digestion, samples were removed from beads and incubated with an additional 500 ng trypsin in 20 mM Tris-HCl (pH 8.0) overnight at 37°C. After protein digestion, samples were acidified to 2% formic acid and one quarter (equivalent to half of one 150 mm plate of the starting cellular material) was taken for MS analysis. Samples were stored at −40°C, if necessary, until analyzed by MS. Samples were always processed in parallel with a negative control (FLAG vector alone).

### Liquid chromatography-MS/MS analysis

Samples were resuspended in 5% formic acid before loading onto fused silica capillary columns (0.75 µm internal diameter) packed in-house with 10 cm Zorbax C18 (ZorbaxSB, 3.5 µm; Sigma-Aldrich, 50254-U) and pre-equilibrated with high-performance liquid chromatography (HPLC) buffer A comprising 3% acetonitrile and 0.1% formic acid. The amount of affinity-purified material loaded on the column was equivalent to two 150 mm plates. Loaded columns were placed in-line with a LTQ mass spectrometer (Thermo Fisher Scientific) equipped with an Agilent 1100 capillary HPLC. The HPLC gradient was delivered at 200 nl/min using a split-flow arrangement. Buffer B was 80% acetonitrile and 0.1% formic acid. The HPLC gradient program delivered an acetonitrile gradient over 120 min (1-5% buffer B over 4 min, 5-40% buffer B over 100 min, 40-60% buffer B over 5 min, 60-100% buffer B over 5 min, hold buffer B at 100% 3 min, and 100-0% buffer B in 2 min). The parameters for data-dependent acquisition on the LTQ mass spectrometer were: one centroid MS (mass range 350-2000) followed by MS/MS on the five most abundantions.

### MS data analysis

All ThermoFinnigan RAW files were saved in our local interaction proteomics laboratory information management system (LIMS), ProHits (Liu et al., 2010). mzXML files were generated from ThermoFinnigan RAW files using the ProteoWizard converter, implemented within ProHits (--filter ‘peakPicking true2’ --filter ‘msLevel2’) (Liu et al., 2010). The searched database contained the human complement of the RefSeq protein database (version 45) (https://www.ncbi.nlm.nih.gov/refseq/) complemented with adenovirus sequences (34,604 entries searched). mzXML files were searched with Mascot version 2.3 (Matrix Science) using the following parameters: one missed cleavage site, methionine oxidation and asparagine/glutamine deamidation as variable modifications. The fragment mass tolerance was 0.6 Da (monoisotopic mass) and the mass window for the precursor was ±3 Da average mass.

### SAINT

Significance analysis of INTeractome (SAINT) (Choi et al., 2011) of affinity-purified samples was done using two biological replicates per bait. Bait protein samples were analyzed alongside eight negative control runs (each treated as a separate bait), consisting of purifications from cells expressing the FLAG tag alone. Individual SAINT scores, indicating the likelihood for a true protein interaction to exist, were first computed for each prey protein in independent biological replicates, before a final SAINT score for each prey protein was calculated as the average of its scores in the two individual replicates (AvgP). SAINT parameters were as follows: eight negative controls compressed into six (nControl=6); nburn=2000, niter=5000, lowMode=0; minFold=1, normalize=1. After SAINT analysis, results were further filtered to show only those proteins identified in with an average SAINT probability of 0.9, at least three spectral counts, and with a frequency of identification less than in 10% of all the samples annotated in a database consisting of >2000 FLAG AP-MS experiments.

### Co-immunoprecipitation

Human HEK293T cells were transfected using Effectene transfection reagent (QIAGEN). Cells were lysed 24-48 h post-transfection in 50 mM Tris pH 7.4, 100 mM EDTA, 150 mM NaCl and 0.5% Triton X-100 and protease inhibitor cocktail (Complete mini EDTA free, Roche) and, to examine Dvl2-OTULIN interaction, 4 μM MG132 (Sigma-Aldrich) was also added. Lysates were pre-cleared with 10 μl of protein A/G plus agarose beads (Santa Cruz Biotechnology) for 30 min with rocking at 4°C. Aliquots containing 400 μg total protein were incubated for 1 h with 1 μg anti-FLAG antibody (mouse M2, Sigma-Aldrich; F1804), 2 μg anti-myc antibody (mouse, Santa Cruz Biotechnology; clone 9E10, sc-40), 1 μg anti-GFP antibody (mouse, Roche; 11814460001) or 2 μg anti-HA antibody (rat, high-affinity, Roche; clone 3F10, 11 867 423 001). 20 μl of protein A/G plus agarose bead slurry was added, incubated for 4 h at 4°C with rocking, and washed thrice with 20 mM HEPES pH 7.5, 500 mM NaCl, 10% glycerol, 0.5% Triton X-100, 1.5 mM MgCl_2_, 10 mM NaPO_4_ pH 7.5 and protease inhibitor cocktail. Samples were analyzed by immunoblotting.

### Testing candidate OTULIN substrates for linear ubiquitination

Transiently transfected HEK293T cells were lysed 24 h post-transfection in 5 M urea, 135 mM NaCl, 1% Triton X-100, 1.5 mM MgCl_2_ and 2 mM N-ethyl maleimide (NEM), supplemented with protease inhibitor cocktail. 300 ml of lysis buffer was added to each well of a six-well plate, and the resulting lysates were sonicated for 20 s at 10% power and centrifuged at 12,000 ***g*** for 20 min. For immunoprecipitation under denaturing conditions, harvested cells were lysed in immunoprecipitation buffer with 1% SDS and 5 mM DTT. Lysates were immunopurified by incubating overnight with GFP-agarose beads (Chromotek) or M2 FLAG-agarose beads (Sigma-Aldrich). After five washes, immunopurified samples were incubated at 70°C for 10 min in LDS dye (Invitrogen) supplemented with fresh 5 mM DTT to elute protein complexes and probed with human anti-linear ubiquitin (gift from Genentech, 1 mg/ml) by immunoblotting. Assays were performed under native and denaturing conditions minimally three times.

### Linear ubiquitin pull-down assays

FLAG-SCRIB, FLAG-NEMO, GFP-VANGL2, Venus-PRICKLE1 or GFP vectors were transiently co-transfected with HA-HOIL and myc-HOIP (Roche, X-tremeGENE) vectors to promote the formation of linear ubiquitin chains in HEK293T cells. Additional co-transfection with FLAG-OTULIN or catalytically attenuated FLAG-OTULIN^C129S^ further tested whether any observed UBAN-GST associations were lost upon Met1-Ub removal. Linear ubiquitin conjugates were isolated from cell lysates using GST alone or GST fused to the WT (UBAN) or mutated (RRE) UBAN region of human NEMO from residues 257-346 (named GST, UBAN-GST and RRE-GST, respectively). Cells were lysed in immunoprecipitation buffer [1% NP-40, 50 mM Tris, pH 7.4, 150 mM NaCl, 0.5% sodium deoxycholate, 2 mM NEM, complete protease inhibitor cocktail (Roche)]. Lysates were sonicated for 20 s at 10% power and centrifuged at 12,000 ***g*** for 20 min at 4°C. The supernatant was precleared with A/G beads for 1 h at room temperature. Supernatants were pre-cleared with sepharose beads (GE Healthcare) for 1 h at 4°C, centrifuged at 500 ***g*** for 5 min to remove sepharose beads and incubated with 120 μg GST, UBAN-GST or RRE-GST glutathione sepharose 4B beads (GE Healthcare) at 4°C overnight under rotation. Beads were washed four times in 500 μl immunoprecipitation buffer. Bound material was eluted with 2× Laemmli buffer. Assays were performed under native and denaturing conditions minimally three times.

### Immunoblotting

Proteins were extracted from MDA-MB-231 cells with TNTE lysis buffer [50 mM Tris-HCl (pH 7.4), 0.5% Triton X-100, 1 mM EDTA, 1× PhosSTOP (Roche, 4906845001) and 1× cOmplete Protease inhibitor cocktail (Roche, 11836153001)]. 15 μg protein from each sample was separated by gradient 10% SDS-PAGE, transferred onto nitrocellulose membranes, probed with primary antibodies (as indicated below) and followed by HRP-linked secondary antibodies, and detected using Amersham ECL Prime Western Blotting Detection Reagent (45002401, GE Healthcare). The antibodies used for immunoblotting were: polyclonal rabbit anti-OTULIN (1:1000, generated in our laboratory; Rivkin, 2010), rat anti-HA (1:3000, high-affinity, Roche; clone 3F10, 11 867 423 001), HRP-linked donkey anti-rabbit (1:10,000, Jackson ImmunoResearch, 711-035-152) and HRP-linked donkey anti-rat (1:10,000, Jackson ImmunoResearch, 712-035-150) antibodies. Anti-tubulin (1:2500, Sigma-Aldrich, T9026) was used as a loading control for the protein input of each lane.

### Biotinylation of cell surface protein

Cell surface proteins were purified from transiently transfected HEK293T cells by use of the Pierce Cell Surface Protein Isolation Kit (Thermo Fisher Scientific) according to the manufacturer's instructions. In brief, cells at 90-95% confluence in a T75 flasks were incubated with 10 ml of sulfo-NHS-biotin solutions (0.25 mg/ml in PBS) for 30 min at 4°C. After the reaction was quenched by 500 µl of quenching solution, cells were scraped, washed with TBS buffer and sonicated. The protein concentration was determined by Bradford Assay. Biotinylated proteins were incubated with Immobilized NeutrAvidin Gel (Pierce), eluted according to the manufacturer's directions and loaded onto SDS-PAGE, and immunoblots were probed with anti-GFP antibodies (1:1000, Roche, 11814460001) to detect GFP-VANGL2. Detection of tubulin with anti-tubulin antibody (1:2500, Sigma-Aldrich, T9026) was used as a threshold above which samples were considered to be contaminated with intracellular proteins. All experiments were done in biological triplicate.

### Immunofluorescence

The primary antibodies used for immunofluorescence were: mouse anti-FLAG M2 (1:1000, Sigma-Aldrich, clone F1804), rat anti-HA (1:200, Sigma-Aldrich, clone 3F10), rabbit anti-myc (1:200, Cell Signaling Technology, 2272), rabbit anti-HOIP (1:200, Abcam, ab125189), rabbit anti-SCRIB (1:200, Abcam, ab36708), mouse anti-SCRIB [1:200, Santa Cruz Biotechnology, Scrib(D2), sc-374139], rabbit anti-VANGL2 [1:200, gift from Dr Matthew Kelley (National Institutes of Health) and Dr Mireille Montcouquiol (INSERM, Université de Bordeaux); used for some embryonic analyses] and rabbit anti-VANGL2 (1:200, Proteintech, 21492-1-AP; used for some embryonic and for all MDA-MB-231 cell analyses). Secondary antibodies were all used at 1:500 dilutions and were: donkey anti-rat Cy3 (712-165-153), donkey anti-mouse Cy3 (715-165-150) from Jackson ImmunoResearch; and donkey anti-rabbit Cy3 (711-165-152), donkey anti-mouse Alexa Fluor 488 (A-11029), donkey anti-rat Alexa Fluor 488 (A-11006) and Alexa Fluor 594 chicken anti-rat (A-11039) from Invitrogen, Molecular Probes. All slides were mounted with Vectashield mounting medium plus DAPI (Vector laboratories). All images were acquired using 60× or 100× oil-immersion objective lens (Nikon D-eclipse C1 confocal microscope system) and analyzed with Adobe Photoshop.

MDCK cells were cultured in 10% FBS/L-glutamate/DMEM, grown for 24 h to 70-80% confluency, fixed with 4% formaldehyde in PBS for 5 min, blocked in 10% normal donkey serum (NDS) and 0.3% Triton X-100 in PBS for 1 h at room temperature, successively incubated with a polyclonal rabbit anti-OTULIN antibody (1:1000; Rivkin et al., 2013) in 0.1% Triton X-100 and 2% NDS in PBS for 1 h at room temperature and then with a Cy3 anti-rabbit antibody, washed, mounted, and imaged.

The number of contacts a given cell made with neighboring cells was counted as were its free edges; a single cell with no contacts was scored as having four free edges. The localization of OTULIN with GFP-VANGL1 or GFP-VANGL2 was assessed by manually counting the number of free edges or cell contacts for each MDCK line (untransduced cells, GFP-VANGL1- and GFP-VANGL2-transduced cells). For each line, the cell surfaces of 50-100 cells were counted for each of three separate experiments. Statistical significance values between the localization of OTULIN in untransduced cells compared that in to VANGL1- or VANGL2-expressing cells were determined using two-way ANOVA and the Bonferroni post test.

HEK293 and Neuro2A cells were fixed 24 h post transfection in 4% formaldehyde in PBS (without Ca^2+^ and Mg^2+^), washed three times for 5 min with PBS, permeabilized with 0.3% Triton X-100 in PBS, washed three times for 2 min in PBS, incubated in blocking buffer (5% BSA, 5% NDS, 5% normal goat serum and 0.1% Triton X-100 in PBS) for 1 h at room temperature, and incubated overnight at 4°C with primary antibodies diluted in blocking buffer. On the next day, cells were washed three times for 5 min in PBS, incubated for 1 h at room temperature with secondary antibodies diluted in blocking buffer, washed three times for 5 min in PBS and mounted with Vectashield mounting medium with DAPI. Images were acquired using 60× oil-immersion objective lens (Nikon D-eclipse C1 confocal microscope system) and analyzed with Adobe Photoshop. All experiments were done at a minimum in biological triplicate. At least 60 cells were analyzed per triplicate.

Immunofluorescence on 12 µm cryosections (acquired using a Leica CM3050S cryostat) of E10.5 embryos was performed essentially as described in Montcouquiol et al. (2003). For anti-VANGL2 immunofluorescence, sections were blocked with 10% BSA and 0.3% Triton X-100 in PBS, incubated overnight in a 1:50 dilution of rabbit anti-VANGL2 antibody (gift from Dr Matthew Kelley and Dr Mireille Montcouquiol) at 4°C. For anti-SCRIB immunofluorescence, sections were incubated overnight with anti-SCRIB antibody (1:200, Abcam, ab36708) in 10% NDS in PBS containing 0.1% Tween 20 (PBS-T). Sections were washed and incubated with secondary anti-rabbit Cy3 antibody for 1 h at room temperature, washed in PBS-T, mounted and imaged. For embryos with closed neural tubes, six embryos of each genotype were analyzed. All embryos with open neural tubes were analyzed.

### Generation of *OTULIN^−/−^* MDA-MB-231 cells via CRISPR

MDA-MB-231 cells were a gift from Dr Stephane Angers (University of Toronto, Canada). Cells were cultured in DMEM with 10% FBS and 100 U/ml penicillin/streptomycin. Cells were maintained at 37°C in a humidified atmosphere containing 5% CO_2_. MDA-MB-231 cells were transfected with all-in-one CRISPR/Cas9 vectors expressing multiple gRNAs with Cas9 using Lipofectamine LTX (Invitrogen) following the manufacturer's instructions. All-in-one CRISPR/Cas9 vectors for the *OTULIN* gene were purchased from Genecopeia and are listed in Table S3. MDA-MB-231 cell clones were selected with 500 µg/ml geneticin (G418, Millipore). Once colonies were obtained, clones were sequence analyzed. Positive CRISPR clones were transfected with HA-VANGL2 using Lipofectamine LTX and analyzed by immunofluorescence.

Candidate clones were amplified by PCR of CRISPR/Cas9-targeted exons from genomic DNA from MDA-MB-231 knockout cells. All clones were verified by sequencing. *OTULIN* clone 60 with a frameshift mutation that led to the premature stop codon formation was used for Wnt5a treatment and future analyses. *OTULIN* clone 25 was used for comparison.

### Analyses of Wnt5a responses

MDA-MB-231 cells were grown to 80-90% confluence, seeded onto sterilized glass coverslips and transfected using Lipofectamine LTX with HA-VANGL2 next day. 23 h post transfection, cells were washed with pre-warmed PBS and starved in DMEM containing 0.2% FBS for 2 h. After starvation, cells were treated for 4 h with conditioned Wnt5a medium (prepared as described in Chen et al., 2003) diluted 1:1 with DMEM containing 10% FBS, with subsequent staining with CellTracker Green CMFDA (2.5 µM, Thermo Fisher Scientific) for 30 min at 37°C. Cells were fixed with 4% formaldehyde and immunofluorescence was performed as described above. All experiments were done in biological triplicate. Cells showing HA-VANGL2 cytoplasmic redistribution or presence of intercellular HA-VANGL2 puncta were scored. Data were analyzed using Student’s one-tailed unpaired *t*-test, type 2 (two-samples; assuming equal variance).

## Supplementary Material

10.1242/dmm.049762_sup1 Supplementary information
