# Supplementary material for: An interaction between OTULIN and SCRIB uncovers roles for linear ubiquitination in planar cell polarity
Source: Dis Model Mech. 2023 Aug 17;16(8):dmm049762. doi: 10.1242/dmm.049762 (PMC10445738; doi:10.1242/dmm.049762)
Supplement: Supplementary information [file dmm-16-049762-s1.pdf]

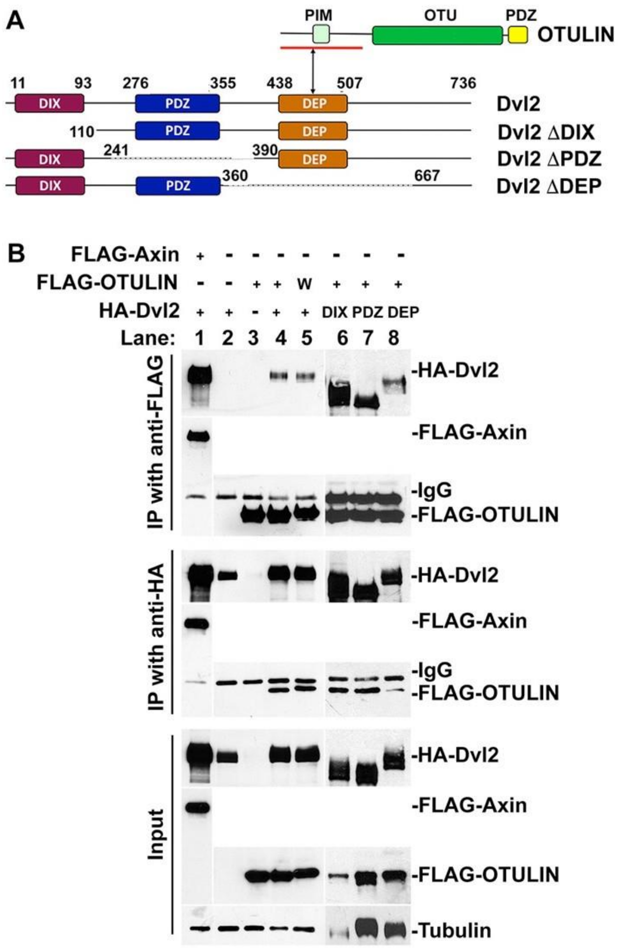

Fig. S1. Optimal OTULIN-Dvl2 interaction requires the PCP signaling associated DEP domain of Dvl2

(A) Schematic diagram of OTULIN and Dvl2 domains, interacting regions, and Dvl2 constructs analyzed. (**B)** Immunoprecipitation (IP) with anti-FLAG antibody of FLAG-Axin, as a positive control, and of all FLAG-OTULIN constructs except for FLAG-OTULIN$^{\Delta 54}$ or FLAG-OTULIN$^{\Delta 54C129S}$ recovers HA-Dvl2, HA-Dvl2 missing the DIX or PDZ domains (Dvl2$^{\Delta DIX}$ and Dvl2$^{\Delta PDZ}$, respectively) equivalently, but FLAG-OTULIN recovers markedly less HA-Dvl2 lacking its DEP domain (Dvl2$^{\Delta DEP}$). Reciprocal co-immunoprecipitation with anti-HA antibody confirmed these interactions and their relative strength. Input amounts and Tubulin levels are shown for all lysates, which were treated with 4uM MG132 for four hours prior to lysis.

Disease Models & Mechanisms • Supplementary information

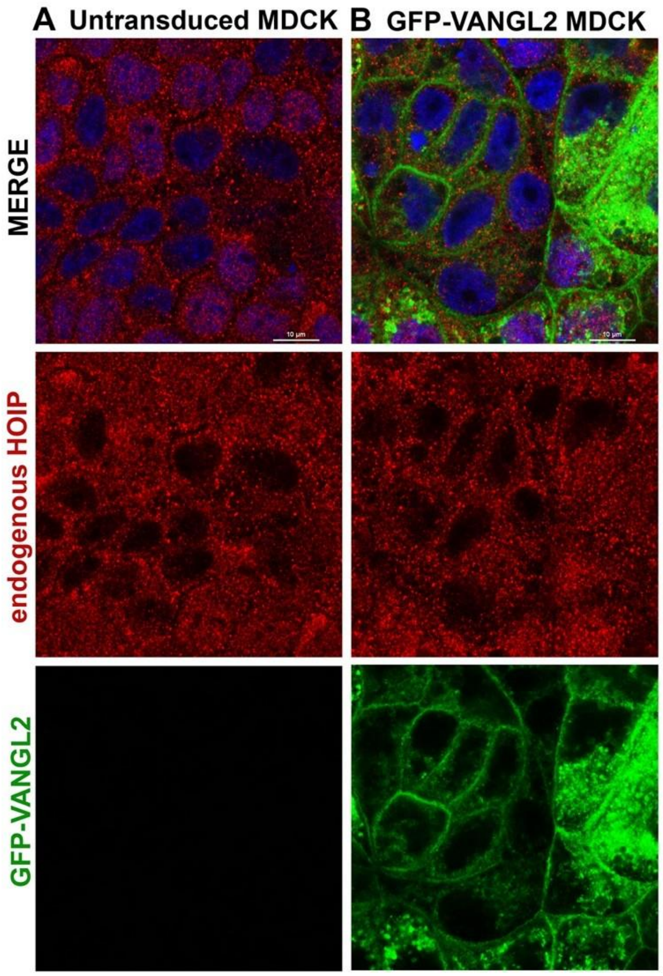

Fig. S2. Localization of endogenous HOIP in untransduced and GFP- VANGL2 transduced MDCK cells.

Immunofluorescent analyses using an anti-HOIP antibody detected endogenous HOIP (red) throughout (**A**) untransduced MDCK and (**B**) GFP-VANGL2 transduced MDCK cells (GFP-VANGL2 MDCK). Images are representative of more than three independently performed experiments. Scale bars indicate 10μm.

Disease Models & Mechanisms • Supplementary information

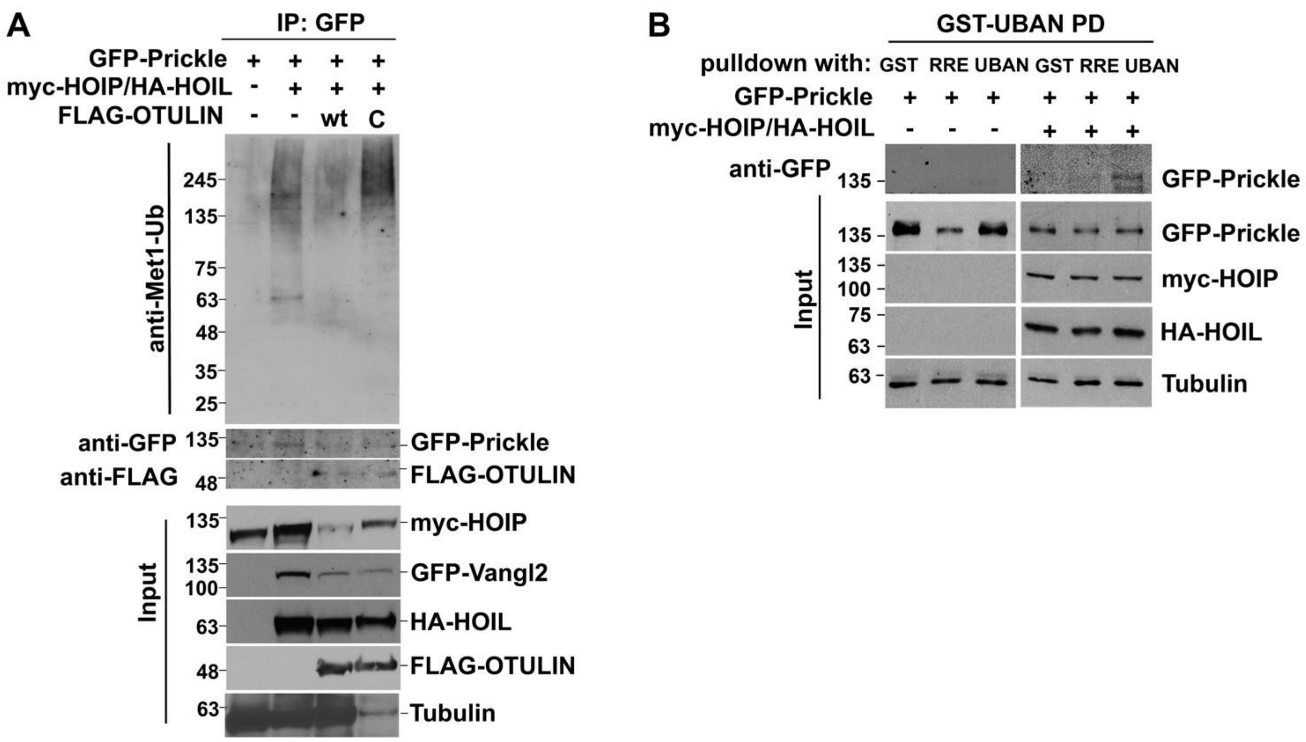

Fig. S3. PRICKLE1 is modified with linear ubiquitin chains.

A. Anti-Met1-Ub antibody detects proteins modified with Met1-Ub chains in immunoprecipitates of GFP-PRICKLE1 recovered with an anti-GFP antibody from HEK293T cells transfected with LUBAC (HA-HOIL and myc-HOIP). Input amounts for each construct and Tubulin levels are shown for each lysate. **B.** Purification of Met1-Ub conjugated GFP-PRICKLE1 using GST-coupled to the Met1-Ub-binding UBAN domain of NEMO (GST-UBAN) or to a mutated non-Met1-Ub binding UBAN domain (GST-RRE). Pull-down with Glutathione Sepharose beads on HEK293T cell lysates transfected with LUBAC (HA-HOIL/myc-HOIP) and input lysates and GST protein added were examined by immunoblotting with anti-GFP antibody and anti-GST antibody. GST-UBAN recovers GFP-PRICKLE1 from LUBAC-expressing lysates.

A

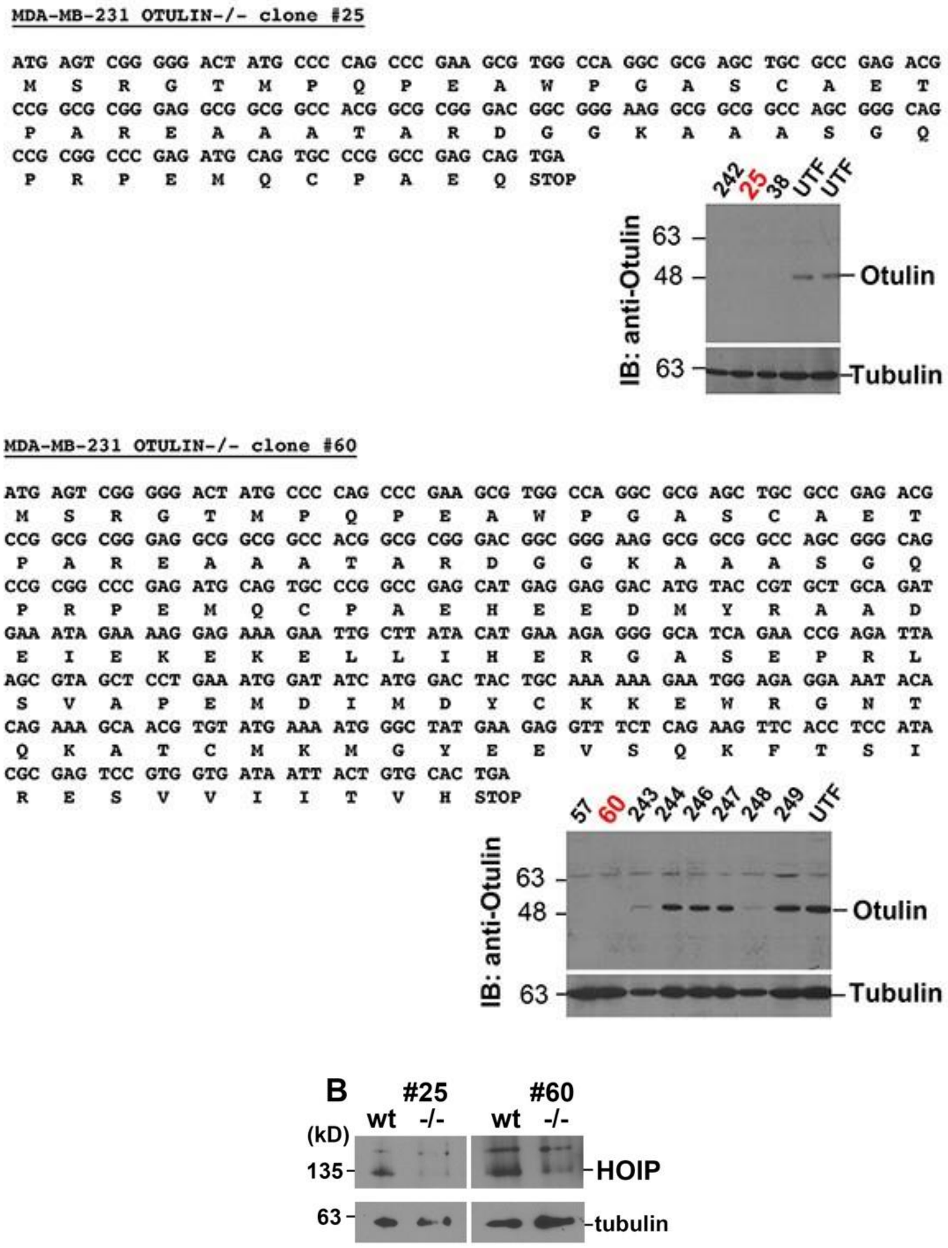

Fig. S4. MDA-MB-231 OTULIN knock-out cells generated by CRISPR cells. OTULIN sequences of MDA-MB-231 cell clones #25 and #60, which were used in parallel for all analyses are shown. Newly generated stop codons in the OTULIN sequence of Clones #25 and #60 were predicted to either lead to nonsense mediated decay or generate truncated inactive OTULIN proteins. No OTULIN protein could be detected in lysates from Clones 25 or 60 by immunoblot with an anti-OTULIN specific antibody. (**B**) Endogenous HOIP levels are reduced in the two MDA-MB- 231 cells lacking OTULIN (#25 and 60) analyzed, as shown in representative immunoblots probed with an antibody against HOIP.

Disease Models & Mechanisms • Supplementary information

Table S1. Summary of statistically significant interactors identified by AP/MS experiments on FLAG-tagged OTULIN constructs.

Protein hits for each FLAG-tagged OTULIN cell line indicated are listed in order of descending statistical significance. Comparison of the profile of significant interactors reveals an interaction with SCRIBBLE occurs through the OTULIN PBM as it is found in wild type OTULIN and OTULIN$^{\Delta 54}$, but not OTULIN$^{\Delta PBM}$ and OTULIN^{C105X} constructs. Only interactors that met a SAINT threshold of >0.8 (highlighted in grey in the 'Avg SAINT' columns) in the AP/MS analysis of at least one FLAG-OTULIN line are listed. Frequently identified proteins (found in >20% of AP/MS projects in the database) are highlighted in grey in the 'Project Frequency' column.

<table>
<tr><th></th><th></th><th></th><th colspan="2">OTULIN</th><th colspan="2">OTULIN Δ54</th><th colspan="2">OTULIN ΔPBM</th><th colspan="2">OTULIN C105X</th></tr>
<tr><th>Hit Gene</th><th>Full name</th><th>Project Frequency</th><th>counts</th><th>Avg SAINT</th><th>counts</th><th>Avg SAINT</th><th>counts</th><th>Avg SAINT</th><th>counts</th><th>Avg SAINT</th></tr>
<tr><td>OTULIN</td><td>Ovarian Tumor Domain proetin with Linear Ubiquitin Specificity</td><td>2.47</td><td>80|23|40|64</td><td>-</td><td>142|150</td><td>-</td><td>67|11</td><td>-</td><td>31|80|50|93</td><td>-</td></tr>
<tr><td>SNX27</td><td>sorting nexin 27</td><td>1.68</td><td>61|26|40|44</td><td>1.00</td><td>174|75</td><td>1.00</td><td>0|0</td><td>0.00</td><td>0|0|0|0</td><td>0.00</td></tr>
<tr><td>HOIP</td><td>ring finger protein 31</td><td>1.19</td><td>27|6|31|15</td><td>1.00</td><td>0|0</td><td>0.00</td><td>23|6</td><td>1.00</td><td>15|18|7|14</td><td>1.00</td></tr>
<tr><td>SCRIB</td><td>scribble</td><td>4.73</td><td>109|16|26|83</td><td>1.00</td><td>2|27</td><td>0.90</td><td>0|0</td><td>0.00</td><td>0|0|0|0</td><td>0.00</td></tr>
<tr><td>SLC9A3R2</td><td>solute carrier family 9, subfamily A member 3 regulator 2</td><td>0.53</td><td>4|0|3|6</td><td>1.00</td><td>0|0</td><td>0.00</td><td>0|0</td><td>0.00</td><td>0|0|0|0</td><td>0.00</td></tr>
<tr><td>CALU</td><td>calumenin</td><td>23.9</td><td>8|0|0|8</td><td>0.92</td><td>0|0</td><td>0.00</td><td>1|1</td><td>0.00</td><td>0|0|4|0</td><td>0.16</td></tr>
<tr><td>CPVL</td><td>carboxypeptidase, vitellogenic-like</td><td>4.9</td><td>3|0|0|2</td><td>0.88</td><td>0|1</td><td>0.00</td><td>0|0</td><td>0.00</td><td>0|0|0|0</td><td>0.00</td></tr>
<tr><td>TIMM50</td><td>translocase of inner mitochondrial membrane 50 homolog</td><td>24.6</td><td>3|2|0|1</td><td>0.88</td><td>0|0</td><td>0.00</td><td>0|0</td><td>0.00</td><td>0|0|1|0</td><td>0.00</td></tr>
<tr><td>TUBA1A</td><td>tubulin, alpha 1a</td><td>18.15</td><td>73|0|25|0</td><td>0.84</td><td>0|0</td><td>0.00</td><td>0|0</td><td>0.00</td><td>30|27|0|0</td><td>0.00</td></tr>
<tr><td>DNAJA2</td><td>DnaJ (Hsp40) homolog, subfamily A, member 2</td><td>27.74</td><td>11|1|2|5</td><td>0.82</td><td>0|0</td><td>0.00</td><td>2|0</td><td>0.00</td><td>1|1|3|4</td><td>0.39</td></tr>
<tr><td>ARL1</td><td>ADP-ribosylation factor-like 1</td><td>9.81</td><td>2|1|0|2</td><td>0.80</td><td>0|0</td><td>0.00</td><td>1|1</td><td>0.00</td><td>0|1</td><td>0.00</td></tr>
<tr><td>SNTB2</td><td>syntrophin, beta 2</td><td>1.24</td><td>0|0|0|0</td><td>0.00</td><td>3|4</td><td>0.98</td><td>0|0</td><td>0.00</td><td>0|0</td><td>0.00</td></tr>
<tr><td>EIF4A2</td><td>eukaryotic translation initiation factor 4A2</td><td>3.27</td><td>0|0|0|0</td><td>0.00</td><td>0|0</td><td>0.00</td><td>7|4</td><td>1.00</td><td>0|4</td><td>0.50</td></tr>
<tr><td>DNM1L</td><td>dynamin 1-like</td><td>4.86</td><td>0|0|0|0</td><td>0.00</td><td>0|0</td><td>0.00</td><td>3|3</td><td>0.99</td><td>0|1</td><td>0.00</td></tr>
<tr><td>DPYSL2</td><td>dihydropyrimidinase-like 2</td><td>6.4</td><td>0|0|0|0</td><td>0.00</td><td>0|0</td><td>0.00</td><td>3|4</td><td>0.98</td><td>0|3</td><td>0.00</td></tr>
<tr><td>TIMM44</td><td>translocase of inner mitochondrial membrane 44 homolog</td><td>6.14</td><td>0|0|0|0</td><td>0.00</td><td>0|0</td><td>0.00</td><td>2|2</td><td>0.80</td><td>0|0</td><td>0.00</td></tr>
<tr><td>PFDN4</td><td>prefoldin subunit 4</td><td>5.21</td><td>0|0|0|0</td><td>0.00</td><td>0|0</td><td>0.00</td><td>2|2</td><td>0.80</td><td>1|0</td><td>0.00</td></tr>
<tr><td>TPT1</td><td>tumor protein, translationally-controlled 1</td><td>2.96</td><td>0|0|0|0</td><td>0.00</td><td>0|0</td><td>0.00</td><td>2|2</td><td>0.78</td><td>0|0</td><td>0.00</td></tr>
<tr><td>GMPS</td><td>guanine monphosphate synthase</td><td>4.73</td><td>0|0|0|0</td><td>0.00</td><td>0|0</td><td>0.00</td><td>2|2</td><td>0.78</td><td>0|0</td><td>0.00</td></tr>
<tr><td>TRAP1</td><td>TNF receptor-associated protein 1</td><td>23.45</td><td>0|0|7|0</td><td>0.08</td><td>0|0</td><td>0.00</td><td>10|11</td><td>0.72</td><td>1|6</td><td>0.03</td></tr>
<tr><td>PAFAH1B2</td><td>platelet-activating factor acetylhydrolase 1b, catalytic subunit 2</td><td>0.75</td><td>0|0|0|0</td><td>0.00</td><td>0|0</td><td>0.00</td><td>0|0</td><td>0.00</td><td>2|2</td><td>0.80</td></tr>
</table>

Disease Models & Mechanisms • Supplementary information

Table S2. Comparative summary of statistically significant OTULIN-interacting proteins of wild type and OTULIN^{W96R} in the presence of the proteasome inhibitor MG132

Treatment of the proteasomal inhibitor MG132 reduced the overall detected interacting proteins for both wild type (WT) and OTULIN^{W96R} cell lines. Proteins identified as meeting an average SAINT statistical cutoff of 0.8 are highlighted in yellow and listed in descending order. SAINT scores highlighted in grey represent interactors that do not meet significance in the indicated cell line although meeting the cutoff in at least one other line. A project frequency of over 20% indicates that the indicated protein, although meeting the SAINT cutoff is found in 1 out of 5 mass spec runs and may represent a false positive.

<table>
<tr><th></th><th></th><th></th><th colspan="2">OTULIN</th><th colspan="2">OTULIN with MG132</th><th colspan="2">OTULIN W96R</th><th colspan="2">OTULIN W96R with MG132</th></tr>
<tr><th>Hit Gene</th><th>Full name</th><th>Project Frequency</th><th>counts</th><th>Avg SAINT</th><th>counts</th><th>Avg SAINT</th><th>counts</th><th>Avg SAINT</th><th>counts</th><th>Avg SAINT</th></tr>
<tr><td>OTULIN</td><td>Ovarian Tumor Domain proetin with Linear Ubiquitin Specificity</td><td>2.47</td><td>80|23|40|64</td><td>-</td><td>57|42|7|15</td><td>-</td><td>13|14|24|17</td><td>-</td><td>2|19</td><td>-</td></tr>
<tr><td>SNX27</td><td>sorting nexin 27</td><td>1.68</td><td>61|26|40|44</td><td>1.00</td><td>123|128|10|27</td><td>1.00</td><td>15|17|20|64</td><td>1.00</td><td>0|30</td><td>0.50</td></tr>
<tr><td>HOIP</td><td>ring finger protein 31</td><td>1.19</td><td>27|6|31|15</td><td>1.00</td><td>0|0|0|7</td><td>0.50</td><td>24|3|18|16</td><td>1.00</td><td>0|8</td><td>0.50</td></tr>
<tr><td>SCRIB</td><td>scribble</td><td>4.73</td><td>109|16|26|83</td><td>1.00</td><td>8|3|2|18</td><td>1.00</td><td>74|24|58|36</td><td>1.00</td><td>0|48</td><td>0.50</td></tr>
<tr><td>SLC9A3R2</td><td>solute carrier family 9, subfamily A member 3 regulator 2</td><td>0.53</td><td>4|0|3|6</td><td>1.00</td><td>0|0|0|0</td><td>0.00</td><td>3|2|0|0</td><td>0.88</td><td>0|1</td><td>0.00</td></tr>
<tr><td>TUBA1C</td><td>tubulin, alpha 1C</td><td>68.73</td><td>89|14|26|47</td><td>0.84</td><td>12|9|8|12</td><td>0.00</td><td>0|18|19|30</td><td>0.00</td><td>89|14|26|47</td><td>0.00</td></tr>
<tr><td>CALU</td><td>calumenin</td><td>23.9</td><td>8|0|0|8</td><td>0.92</td><td>0|0|0|2</td><td>0.03</td><td>6|0|3|4</td><td>0.51</td><td>8|0|0|8</td><td>0.00</td></tr>
<tr><td>CPVL</td><td>carboxypeptidase, vitellogenic-like</td><td>4.9</td><td>3|0|0|2</td><td>0.88</td><td>0|0|0|0</td><td>0.00</td><td>2|2|0|0</td><td>0.80</td><td>3|0|0|2</td><td>0.00</td></tr>
<tr><td>TIMM50</td><td>translocase of inner mitochondrial membrane 50 homolog</td><td>24.6</td><td>3|2|0|1</td><td>0.88</td><td>0|0|1|0</td><td>0.00</td><td>0|1|0|1</td><td>0.00</td><td>3|2|0|1</td><td>0.00</td></tr>
<tr><td>TUBA1A</td><td>tubulin, alpha 1a</td><td>18.15</td><td>73|0|25|0</td><td>0.84</td><td>0|0|0|10</td><td>0.03</td><td>46|0|0|28</td><td>0.82</td><td>73|0|25|0</td><td>0.34</td></tr>
<tr><td>DNAJA2</td><td>DnaJ (Hsp40) homolog, subfamily A, member 2</td><td>27.74</td><td>11|1|2|5</td><td>0.82</td><td>1|0|0|1</td><td>0.00</td><td>3|1|3|4</td><td>0.39</td><td>11|1|2|5</td><td>0.18</td></tr>
<tr><td>ARL1</td><td>ADP-ribosylation factor-like 1</td><td>9.81</td><td>2|1|0|2</td><td>0.80</td><td>0|0|0|0</td><td>0.00</td><td>4|0|0|0</td><td>0.50</td><td>2|1|0|2</td><td>0.00</td></tr>
<tr><td>ATP2A2</td><td>syntrophin, beta 2</td><td>12.28</td><td>1|0|0|1</td><td>0.00</td><td>0|0|0|0</td><td>0.00</td><td>8|0|0|2</td><td>0.89</td><td>1|0|0|1</td><td>0.00</td></tr>
<tr><td>IRS4</td><td>eukaryotic translation initiation factor 4A2</td><td>25.53</td><td>2|0|0|0</td><td>0.00</td><td>0|0|0|0</td><td>0.00</td><td>4|0|0|2</td><td>0.89</td><td>2|0|0|0</td><td>0.00</td></tr>
</table>

Disease Models & Mechanisms • Supplementary information

Table S3. Plasmids used in this study

Construct	Source
HA-OTULIN<br>HA-OTULIN$^{\Delta 54}$<br>HA-OTULIN$^{\Delta PBM}$<br>HA-OTULIN^{C105X}<br>HA- OTULIN^{C129S}<br><br>FLAG-tagged constructs in FLAG-pcDNA3.1 and FLAG-pcDNA5-FRT/TO :<br>FLAG-OTULIN<br>FLAG-OTULIN$^{\Delta 54}$<br>FLAG-OTULIN$^{\Delta PBM}$<br>FLAG-OTULIN^{C105X}<br>FLAG-OTULIN^{W96R}<br>FLAG-OTULIN^{C129S}	described previously in Rivkin et al.,2013.
HA-OTULIN^{Y56A}	generated for this study using QuikChange (Agilent), HA-OTULIN-pcDNA3, and the following oligonucleotides: 5'-GAGCATGAGGAGGACATGGCCCGTGCTGCAGATGAAAT-3' and 5'-ATTTCATCTGCAGCACGGGCCATGTCCTCCTCATGCTC -3'.
Myc-HOIP<br>HA-HOIL<br>FLAG-NEMO	Dr. Kazuhiro Iwai (Kyoto University)
GST-UBAN	Dr. Mads Gryd-Hansen (Oxford University)
GST-RRE	Dr. Jae U. Jung (University of Southern California)
GFP-VANGL2<br>Venus-PRICKLE1<br>FLAG-SCRIB<br>FLAG-Axin<br>HA-Dvl2	Dr. Stephane Angers (Donnelly Centre, University of Toronto)
HA-Dvl2$^{\Delta DIX.}$<br>HA-Dvl2$^{\Delta PDZ}$<br>HA-Dvl2$^{\Delta DEP}$	Dr. Sheng-Cai Lin **(**National University of Singapore)
All-in-one *OTULIN* CRISPR-Cas9 plasmids:<br>HCP221899-CG01-3-B-a,<br>HCP221899-CG01-3-B -b<br>HCP221899-CG01-3-B -c	purchased from GeneCopoeia.
FLAG-pcDNA5-FRT/TO<br>FLAG-pcDNA3.1	OpenFreezer, Network Biology Collaborative Centre (NBCC), Lunenfeld-Tanenbaum Research Institute, Toronto
